# Noninvasive Brain Stimulation in Primary Progressive Aphasia with and Without Concomitant Speech and Language Therapy: Systematic Review and Meta-analysis

**DOI:** 10.1007/s11065-025-09659-5

**Published:** 2025-02-01

**Authors:** Francesco Lomi, Ilaria Simonelli, Stefano Cappa, Patrizio Pasqualetti, Simone Rossi

**Affiliations:** 1 Siena Brain Investigation & Neuromodulation Lab (Si-BIN Lab), Unit of Neurology and Clinical Neurophysiology, Department of Medicine, Surgery and Neuroscience, University of Siena - Policlinico Le Scotte, Viale Mario Bracci, 16, 53100 Siena, Italy; 2 Biostatistics Service, Clinical Research Center, Isola Tiberina-Gemelli Isola Hospital, Via Di Ponte Quattro Capi, 39, Rome, Italy; 3 Department of Biomedicine and Prevention, University of Rome Tor Vergata, Viale Montpellier 1, Rome, Italy; 4 Institute for Advanced Study, IUSS, Piazza Della Vittoria, 15, Pavia, Italy; 5 IRCCS Istituto Auxologico Italiano, Via Magnasco, 2, Milan, Italy; 6 Section of Health Statistics and Biometry, Department of Public Health and Infectious Diseases, Faculty of Pharmacy and Medicine, Sapienza University of Rome, Piazzale Aldo Moro, 5, Rome, Italy

**Keywords:** Noninvasive brain stimulation (NiBS), Transcranial direct current stimulation (tDCS), Transcranial magnetic stimulation (TMS), Primary progressive aphasia, Speech and language therapy

## Abstract

Primary progressive aphasias (PPA) represent a group of neurodegenerative conditions affecting verbal communication abilities for which no effective medication is currently available. Noninvasive brain stimulation (NiBS) has been mainly explored as adjunctive therapy to conventional speech and language therapy (SLT) with promising results. The present meta-analysis of randomized-controlled trials (RCTs) aims to evaluate the efficacy of NiBS in PPA patients on a range of linguistic tasks (naming, phonemic fluency, semantic fluency). A literature search was carried out using EMBASE and PUBMED, searching for multi-session RCTs administering NiBS on PPA patients as stand-alone or with SLT. The results were not significant overall, indicating a null difference between the active and the sham condition on language functions; pooled effects tended to be higher in parallel than in crossover studies and for follow-ups than post-treatment. In the naming analyses, the combined effects for the studies that coupled NiBS with SLT were slightly higher than the overall effect at each time point, although not significant. These results need to be considered with caution given the low number of included studies and small sample sizes, but offer relevant indications for future research in terms of optimal treatment protocols and personalization of therapies.

**Supplementary Information:**

The online version contains supplementary material available at 10.1007/s11065-025-09659-5.

## Introduction

The term primary progressive aphasia (PPA) encompasses a heterogeneous group of disorders characterized by a relatively focal degeneration of the brain language networks (Gorno-Tempini et al., 2011; Marshall et al., 2018). A PPA clinical diagnosis requires a prominent and isolated language deficit during the initial phase of the disease (Gorno-Tempini et al., 2011). This onset is insidious and then the progression of the disease leads to a general impoverishment of the linguistic functions and the involvement of other cognitive domains.

The specificity of the clinical symptoms and involved networks enables us to distinguish, at the relatively early stages of the disease, three different PPA phenotypes (Gorno-Tempini et al., 2011; Mesulam et al., 2021): nonfluent/agrammatic (nfvPPA), logopenic (lvPPA), and semantic (svPPA) variants. From a clinical standpoint, the core features of nfvPPA are the motor speech disorder (apraxia of speech) and/or the loss of grammatical structure of utterances (agrammatism); lvPPA is typically characterized by impaired sentence repetition and single word retrieval; svPPA presents instead with the loss of semantic knowledge that manifests with anomia and deficits in single-word comprehension (Gorno-Tempini et al., 2011). However, mixed and unclassifiable cases are also found in clinical practice (Utianski et al., 2019). Lateralization of the degeneration in the language-dominant hemisphere is observed in all PPA variants at the earlier disease stages, although each clinical subtype is characterized by the involvement of different linguistic areas and circuits (Mesulam et al., 2021). In nfvPPA, degeneration initially involves the left inferior frontal gyrus and the left dorsal premotor and supplementary motor cortices (Grossman, 2010; Grossman et al., 2023; Mandelli et al., 2016), whereas lvPPA and svPPA early neuropathological changes occur in the left inferior parietal and temporoparietal junction regions and the left anterior temporal lobe, respectively (Mandelli et al., 2023; Mesulam et al., 2023). The PPA variants are probabilistically associated with different neuropathological substrates. Most svPPA cases are caused by frontotemporal lobar degeneration (FTLD) with transactive response DNA-binding protein (TDP), while nfvPPA is typically associated with FTLD-tau. LvPPA, on the other hand, is mostly caused by Alzheimer’s disease (AD) pathology (Spinelli et al., 2017).

Epidemiological data on PPA is scarce, and data regarding the real prevalence and incidence of each PPA variant is not available, but an estimate can be derived from epidemiological data of FTLD. The prevalence of the FLTD disorders taken together has been recently estimated to be ~ 10–15 cases per 100,000 (Coyle-Gilchrist et al., 2016; Kvello-Alme et al., 2019), and similar rates have been reported for incidence after 65 years (Logroscino et al., 2019). In autopsy studies, up to 45% of FTLD cases had PPA and nearly half of those PPA patients had nfvPPA (Grossman, 2012). Regarding AD, prevalence is estimated to be ~ 65/100,000 in the 45–64 age range (Kvello-Alme et al., 2019), and approximately 7–9% of AD patients present with language difficulties (Graff-Radford et al., 2021). Survival following diagnosis is about 5 years for nfvPPA and 9 years for svPPA (Coyle-Gilchrist et al., 2016), whereas survival data in lvPPA variant is scarce (Borroni et al., 2022; Graff-Radford et al., 2021).

From a therapeutic point of view, no effective pharmacotherapy exists for the treatment of PPA and only supportive therapies (i.e., speech and language therapy, SLT) are currently adopted to alleviate the symptoms of this disorder (Cotelli et al., 2020; Volkmer et al., 2020). Therefore, research for new therapeutic approaches in this field is urgently needed, also considering that frontotemporal dementia is one of the most prevalent early-onset (< 65 years) dementia subtypes (Vieira et al., 2013), with devastating implications for family life, work, and social functioning.

Hope in this sense is emerging thanks to noninvasive brain stimulation (NiBS) techniques that rely on electromagnetic principles to noninvasively influence neural activity through the induction of electrical fields in the brain. In this way, controllable and long-lasting neuroplasticity changes can be induced in targeted networks (Cirillo et al., 2017). Among these, transcranial direct current stimulation (tDCS) induces a low-amplitude (0.5–2 mA) direct current able to modulate brain excitability in a polarity-specific manner, making neurons more (anodal tDCS) or less (cathodal tDCS) susceptible to membrane depolarization (Paulus, 2003). Other transcranial electrical current techniques deliver sinusoidal currents oscillating at a fixed frequency (transcranial alternating current stimulation, tACS) or randomly within a specific range (transcranial random noise stimulation, tRNS) (Antal & Herrmann, 2016). Transcranial magnetic stimulation (TMS), instead, is based on the creation of a rapidly alternating magnetic field that induces electrical currents in the targeted brain area; these electric fields, differently from the ones mentioned before, are strong enough to generate action potentials in stimulated neurons (Di Lazzaro & Falato, 2020; Rotenberg et al., 2014). Repetitive TMS (rTMS), consisting of trains of pulses delivered at a specific frequency, is able to induce therapeutic neuromodulatory effects (Rossi et al., 2009). Low-frequency repetitive TMS (typically 1 Hz) leads to a suppression of cortical activity in the stimulation location (R. Chen et al., 1997), whereas high-frequency rTMS (typically > 5 Hz) leads to an increase in cortical activity in the stimulated site (Oberman, 2014).

Although the physiological mechanisms underlying neuromodulatory effects of NiBS are complex and need to be further elucidated, probable candidates include long-term potentiation (LTP)/depression (LTD) of synaptic transmission, modifications in protein synthesis (e.g., neurotrophic factors) and glial function (Cirillo et al., 2017). These changes at the microscopical level are mirrored by functional connectivity changes at the network level (J. Chen et al., 2022; Fox et al., 2012; Hordacre et al., 2018; Reed & Cohen Kadosh, 2018).

Both repetitive transcranial magnetic stimulation (rTMS) and tDCS have been successfully used to treat various neurological and psychiatric disorders (Lefaucheur et al., 2017, 2020). A recent meta-analysis suggests that both high-frequency and low-frequency rTMS, especially when applied in multiple sessions, ameliorate cognitive symptoms in Alzheimer’s disease patients, with medium to large effect sizes (Chou et al., 2020). The after-effect of five or more sessions of rTMS could last from a few weeks to 3 months. Noninvasive brain stimulation techniques seem promising also in other dementias (Sanches et al., 2020).

Regarding PPA, these methods have been explored both as stand-alone and as adjunctive therapies to conventional SLT, as pointed out by recent meta-analytical studies (Byeon, 2020; Cotelli et al., 2020; Nissim et al., 2020). The rationale behind this coupling is to promote the learning mechanisms induced by SLT because the brain regions that are stimulated are effectively engaged by speech and language training, with the aim to contrast the worsening of symptoms due to neurodegeneration. This approach has been already followed, with promising results, for rehabilitation after stroke (Baker et al., 2010; Fridriksson et al., 2011; Lefaucheur et al., 2020). However, the previous meta-analyses on NiBS in PPA (Byeon, 2020; Cotelli et al., 2020; Nissim et al., 2020) were limited by the paucity of randomized clinical trials (RCTs) that represent the gold standard for informing on the efficacy of clinical interventions. Since then, few other randomized, sham-controlled studies have been published, so that an updated synthesis of the efficacy of NiBS in this neurodegenerative condition is urgently needed.

Hence, the first aim of the present meta-analysis is, in general, to contribute to the debate on the efficacy of NiBS on dementia, by providing summary data from scientifically rigorous studies on the capacity of these methods to improve linguistic abilities in primary progressive aphasias. A secondary aim, provided that enough studies are available, is to investigate the effect of variables (i.e., presence of SLT, PPA variant, disease severity, stimulation method) that might influence the results, thus informing the scientific community about the most promising protocols to apply for these kinds of patients. We hypothesize that, when coupled with an active training like SLT, NiBS protocols could exert a greater effect on language functions than when are used as stand-alone (Miniussi & Rossini, 2011). In the second instance, given that clinical characteristics and patterns of brain atrophy differ across PPA variants, we might expect that patients with different variants respond differently to the stimulation of the same language hub. Third, the severity of deficits at baseline might be a significant determinant of which PPA patients would benefit from neuromodulation treatments; NiBS could be more beneficial in early disease stages (Bagattini et al., 2020; Cotelli et al., 2016), whereas one study found a larger improvement in advanced PPA stages (McConathey et al., 2017). In the fourth place, we aim to investigate the role of the stimulation method, since previous meta-analyses on Alzheimer’s disease patients suggested differential effects of tDCS and rTMS on cognitive symptoms (Šimko et al., 2022; Teselink et al., 2021), probably due to their differential effect at the neural level as previously described; the inclusion of different NiBS approaches in the same meta-analysis is motivated by their overarching goal of counteracting disease progression via cortical excitability modulation, with the adjunctive effect of SLT.

## Methods

The meta-analysis is reported according to the Preferred Reporting Items for Systematic reviews and Meta-Analyses (PRISMA) (Liberati et al., 2009; Page, McKenzie, et al., 2021; Page, Moher, et al., 2021) (see Table S1 in supplementary materials).

### Eligibility Criteria

The following inclusion criteria were used to screen articles for the meta-analysis: (a) Patients (≥ 5) with a PPA formal diagnosis according to the consensus criteria established by Gorno-Tempini and colleagues (Gorno-Tempini et al., 2011); (b) Noninvasive brain stimulation treatment (e.g., rTMS, tDCS, tACS), alone or in combination with SLT, repeated for at least two sessions; (c) randomized sham-controlled clinical trial (parallel, crossover or mixed); (d) language outcomes measured with standardized or experimental tasks; (e) enough statistical information, such as means (*M*) or medians, standard deviations (SD) or ranges, or *t* or *F*, in order to calculate the effect size and perform the meta-analysis. No restriction on the year of publication, language of publication, or publication status (such as inclusion of unpublished material and abstracts) was considered, provided that the inclusion criteria were satisfied.

We arbitrarily decided to consider only sample sizes including at least five patients, as a way to minimize the possible influence of outliers on results. We then set the cut-off of two stimulation sessions for two main reasons: to rule out potential carryover effects in single-session experimental cross-over studies, especially when stimulation conditions were performed on the same day; to investigate the association between NiBS and SLT, which typically entails multiple sessions.

### Information Sources

The search of the relevant literature was performed via EMBASE and MEDLINE (PubMed) electronic databases, in order to find potentially relevant articles for the meta-analysis. The references of the retrieved papers were also checked to search for additional studies that could be included. The first systematic search was conducted in February 2023, and the last update was carried out in December 2023.

### Search

We used the following search terms, both as keywords and words contained in the title and/or abstract, for the identification of relevant articles: (PPA OR “primary progressive aphasia” OR “neurodegenerative aphasia” OR lvPPA OR “logopenic PPA” OR “logopenic variant” OR nfvPPA OR “non fluent PPA” OR “non fluent variant” OR “non-fluent variant” OR “non-fluent PPA” OR “apraxia of speech” OR AOS OR svPPA OR “semantic PPA” OR “semantic variant” OR “semantic dementia” OR avPPA OR “agrammatic PPA” OR “agrammatic variant”) AND (“Transcranial Magnetic Stimulation” OR “TMS” OR “theta burst stimulation” OR “transcranial direct current stimulation” OR “tDCS” OR “transcranial alternating current stimulation” OR “tACS” OR “transcranial random noise stimulation” OR “tRNS” OR “transcranial electrical stimulation” OR tES OR “electrical stimulation” OR “neuromodulation).

### Study Selection

Study selection was performed independently by the first two authors, and disagreements were resolved by discussion and consensus between the two review authors; if no agreement could be reached, the decision was taken by the third author.

The title and abstract of papers were screened in the first place, and those papers which seemed suitable were then inspected more in-depth by examining the full text. When the full-text article was not found, the corresponding author was contacted to obtain it.

When articles clearly included, at least in part, the same participants (as in the case, for example, of multiple reports from the same study), only the most recent paper was included to avoid dependency biases. When this was not explicitly declared in the full text, but there was a valid reason to suspect the overlapping of participants (e.g., papers conducted by the same group of authors), the corresponding authors were contacted to obtain this information. If the studies were carried out with different participants, they were included in the analyses as independent; in case of same participants or no response by authors, only the more recent paper was included.

### Data Collection Process

The second author developed a data extraction sheet, the first author extracted the data on language measures from included studies, and the second author checked the extracted data. Disagreements were resolved by discussion between the two authors. The data extracted included sample size, *M* and SD, separately for the active and the sham group or condition, or *t* or *F* statistics, so that effect sizes could be calculated. A second data extraction sheet was created by the first author that included descriptive characteristics of the studies together with demographic and additional clinical data of participants.

When the paper or abstract did not include all the necessary data to estimate the study effect size, for example, in the case of unpublished nonsignificant data or composite scores, the corresponding authors were contacted to obtain the missing data. Some authors provided us with summary data (*M* and SD) for each condition and time point, while others sent us the individual scores of the whole sample. When reported in the form of plots, the data was extracted by using WebPlotDigitizer software (Rohatgi, 2024). The raw data obtained by the authors allowed us to calculate *M* and SD for each outcome of interest. Authors were also contacted when relevant demographic, clinical data, and/or information about task design (e.g., number of letters/categories tested in fluency tests, total number of naming items) was missing.

### Data Items

The outcomes considered for this review are the scores on language tests or batteries investigating linguistic processes (e.g., naming, repetition, grammatical comprehension), and scores related to functional communication scales. The outcomes were analyzed if reported by enough included studies (i.e., at least five).

The following data regarding included studies was extracted: (a) design; (b) type of language training (if present); (c) number of sessions; (d) stimulation protocol details; (e) age, sex, and education years; (f) sample size, both overall and separately for each condition (active, sham) and PPA variant (non-fluent, semantic, logopenic). The following clinical variables were also extracted: (g) the FTLD-modified Clinical Dementia Rating total and language score (Knopman et al., 2008); (h) Mini-Mental State Examination (MMSE) score (Folstein et al., 1975); (i) the Montreal Cognitive Assessment (MoCA) score (Nasreddine et al., 2005); (j) years post-onset; (k) age at onset.

Finally, for each outcome of interest, the following data was extracted separately for the active and sham group/condition: (l) *M* and SD at pre-treatment; (m) *M* and SD at post-treatment; (n) *M* and SD of the change between pre- and post-treatment; (o) *M* and SD at follow-up(s); (p) *M* and *SD* of the change between pre-treatment and each follow-up; (q) *t* or *F* statistics of the change between pre- and post-treatment, pre-treatment and first follow-up, and pre-treatment and second follow-up; (r) time interval between assessments.

### Risk of Bias in Individual Studies

The validity of the included studies was evaluated by using the revised Cochrane risk-of-bias tool for randomized trials (RoB-2) (Sterne et al., 2019). This tool evaluates the risk of bias arising from several domains: the randomization process, with questions about the appropriate generation of random allocation sequence and allocation concealment; deviations from intended interventions, assessing crucial aspects like blinding of participants, carers, and health care providers, but also the possibility of imbalance in non-protocol interventions; the presence of missing outcome data; the measurement of the outcome, evaluating the validity of the measurement method and the blinding of outcome assessors; the selection of reported results. For crossover trials, the RoB-2 also includes questions assessing the bias arising from period and carryover effects. The user can choose among five possible answers to each question: “Yes,” “Probably yes,” “Probably no,” “No,” or “No information.” An algorithm assigns a certain risk of bias to each domain based on the answers to individual questions. The possible risk-of-bias judgments are the following: “Low risk of bias,” “Some concerns,” and “High risk of bias.”. If the study is judged to be at low risk of bias for all domains for a specific outcome, it will achieve an overall judgment of “Low risk of bias” for that result. In the case of “Some concerns” in at least one domain, the study will be considered as raising “Some concerns” overall. If a study obtains a “High risk of bias” judgment, or multiple “Some concerns” judgments in a way that substantially lowers confidence in the result, it will obtain an overall judgment of “High risk of bias.”

### Effect Measures

Being the outcome variables continuous, and since the included studies used different scales of measurement, we planned to analyze the difference in variation between intervention and control groups in terms of standardized mean difference (SMD) for all the outcomes (percentage of correct answers in naming, phonemic, and semantic fluency) (Borenstein et al., 2009; Higgins & Green, 2011).

### Synthesis Methods

The data collected from studies was prepared to make it suitable to be included in the synthesis. Regarding the naming outcome, we decided to take the percentage of correct answers (i.e., accuracy) as the measure of interest to make the studies more comparable, given that the number of items can differ across studies. Hence, for the studies that did not report it, the mean percentage of naming accuracy was calculated by dividing the mean number of correct answers by the total number of items. In the same way, the standard deviation of naming accuracy was calculated by dividing the standard deviation of correct answers by the total number of items.

When the data were presented as *M* and standard error (SE), the SE was converted to standard deviation with the formula: $\mathrm{SD}=\mathrm{SE}\times \sqrt{n}$.

When not already reported by the original study, the mean change and relative SD in each group were calculated.

The first was computed as the difference between post-intervention mean and baseline mean: ${M}_{\mathrm{D}}={M}_{\mathrm{B}}-{M}_{\mathrm{P}}$, where ${M}_{\mathrm{B}}$ is the baseline mean and ${M}_{\mathrm{P}}$ is the post-intervention (or follow-up) mean. Regarding the standard deviation of the changes, it was imputed using a correlation coefficient following the method suggested in the *Cochrane Handbook for Systematic Reviews of Interventions* (Higgins & Green, 2011). About naming, the correlation coefficient between pre-treatment data and immediate post-treatment data and at the various follow-ups was calculated from data reported by one included study (Huang et al., 2023), so that SDs for the change for the other studies could be imputed. For the other two outcomes, the correlation between pre-post data in each group was not available; hence, it was calculated from data; for phonemic, we assumed a correlation of 0.86, and for semantic, a correlation of 0.9.

For parallel studies, the standardized mean difference (Cohen’s *d*) was calculated as $d= \frac{{\underline{X}}_{1}- {\underline{X}}_{2}}{S}$, where ${\underline{X}}_{1}$ and ${\underline{X}}_{2}$ are the sample mean change of the two groups, and $S$ is the within-groups standard deviation obtained by pooling the two sample estimates of the SD (Borenstein et al., 2009); for crossover studies, as suggested by the *Cochrane Handbook for Systematic Reviews of Interventions*, $S$ represents the standard deviation of measurements based on a correlation coefficient.

*SMD* values were interpreted as follows: 0.2 represents a small effect, 0.5 a moderate effect, and 0.8 a large effect (Cohen, 1988).

The results of the main analyses were represented graphically by forest plots. We decided to carry out the meta-analyses only when at least five studies were reported for a given outcome, since statistical testing for heterogeneity is not recommended with less than five studies (Schriger et al., 2010).

For each outcome, a random-effects model was performed to calculate the overall *SMD*, which can be expected to incorporate heterogeneity among studies. DerSimonian-Laird random-effects variance estimator was used. The 95% confidence interval (95% *CI*) around the pooled estimated effect was reported. The extent and impact of between-study heterogeneity were assessed by inspecting the forest plots and quantified by calculating the ${I}^{2}$ statistic. The ${I}^{2}$ describes the rate of variation across studies due to heterogeneity rather than chance, ranging from 0 (no heterogeneity) to 100 (maximal heterogeneity) (Higgins et al., 2003). Also the *Q* statistic, along with its degrees of freedom, *p*-value, and tau or tau-squared estimates were reported.

#### Subgroup Analysis and Investigation of Heterogeneity

Possible causes of variation of results across the studies were explored by performing subgroup/meta-regression analyses based on:

- RoB-2 score (low, some concerns, high)
- Disease severity (MMSE score)
- Stimulation type (tDCS, rTMS)
- PPA variant (nfvPPA, lvPPA, svPPA)

The analyses were carried out if there were at least 10 studies (10 studies for each category in case of a categorical variable), as recommended by the Cochrane guidelines (Higgins & Green, 2011).

#### Sensitivity Analysis

A sensitivity analysis was performed excluding studies that did not perform SLT.

#### Reporting Bias Assessment

To assess small-study effects, we planned to generate the funnel plot for the meta-analyses including enough trials of varying size (~ 10) (Higgins & Green, 2011). If asymmetry in the funnel plot was detected, we planned to perform Egger’s test and the trim-and-fill procedure (Duval & Tweedie, 2000).

## Results

### Study Selection

**Fig. 1:**
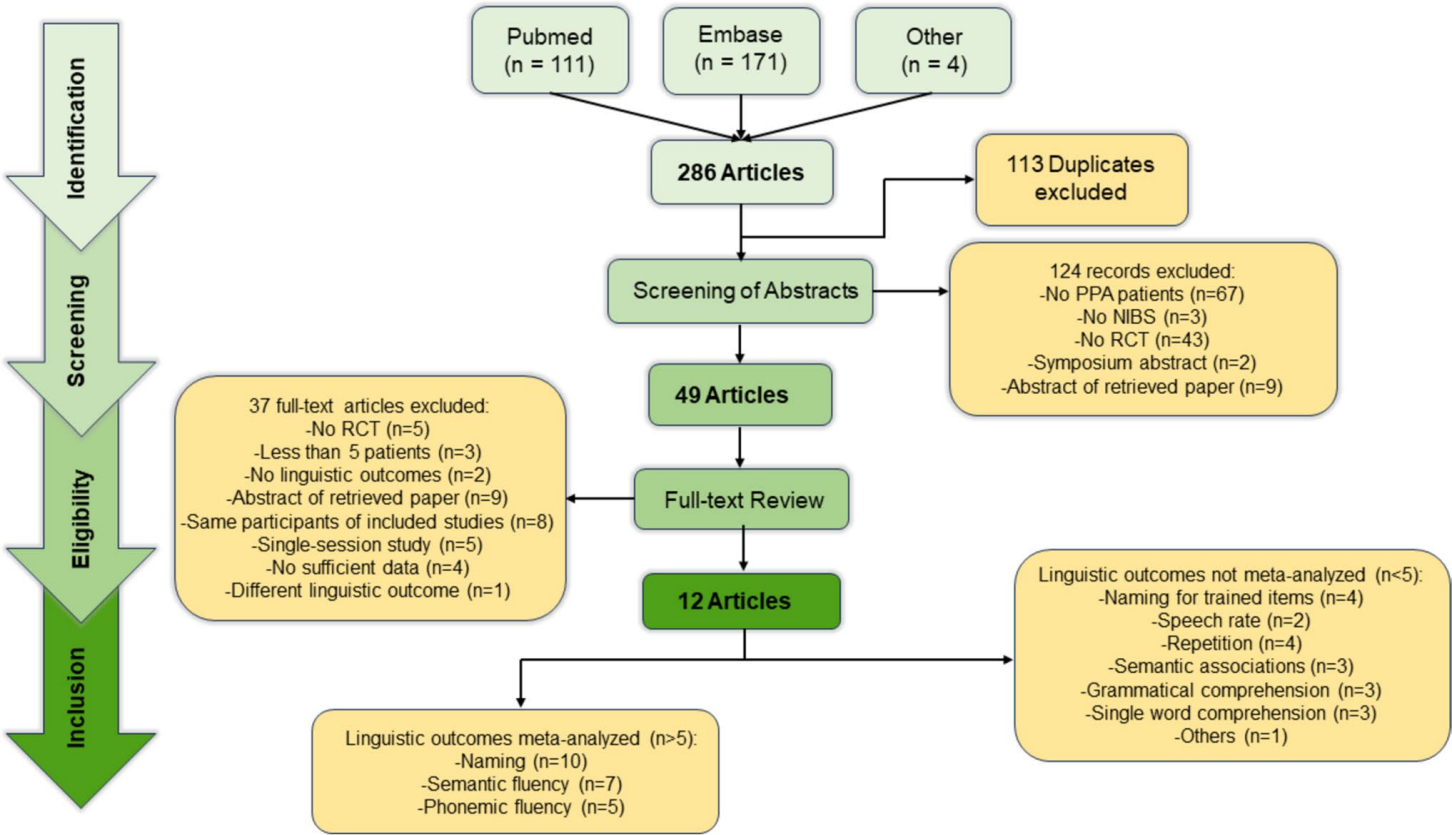
PRISMA flow diagram of the study selection process

The literature search via electronic databases resulted in 282 records, 171 on EMBASE, and 111 on PubMed (see Fig. 1). Four articles were also retrieved by inspecting the references of articles, for a total amount of 286 papers. After adjusting for duplicates, 173 papers remained. Of these, 49 papers passed the first title-and-abstract screening and were examined in more detail to assess their eligibility. A total number of 13 papers were eligible; after the exclusion of one paper, due to different linguistic measures, 12 papers were finally included in the quantitative analysis.

Some preliminary considerations regarding the study selection process are necessary:

- We asked the authors if the retrieved papers by Nissim et al. (2022), Hosseini et al. (2019), and McConathey et al. (2017) did contain, at least in part, the same participants, due to the overlapping authors and research groups (Hosseini et al., 2019; McConathey et al., 2017; Nissim et al., 2022). Since the authors confirmed that there was no overlap of participants, all three studies were included in the meta-analysis.
- We retrieved two studies by the same group of authors (Roncero et al., 2017, 2019). Given the suspect that they might include the same participants, we only included the 2017 paper in the meta-analysis.
- We retrieved 12 articles by the same group of authors that probably included, at least in part, the same cohort of participants (de Aguiar et al., 2020a, 2020b; de Aguiar et al., 2020a, 2020b; Fenner et al., 2019; Ficek et al., 2019; Harris et al., 2019; Herrmann et al., 2022; Licata et al., 2023; Tang et al., 2022; Tao et al., 2021; Tsapkini et al., 2014, 2018; Wang et al., 2022). Indeed, most articles are reports from the same trial, as stated in the full text of the papers. Therefore, we decided to include only the most recent article for which linguistic data were available, which was the paper by Wang and colleagues (Wang et al., 2022) regarding phonemic and semantic fluency outcomes and the one by De Aguiar and collaborators (de Aguiar et al., 2020a, 2020b) regarding the naming outcome. The latter was actually preferred over a more recent paper (Tao et al., 2021) because of a considerably larger sample size.

### Study Characteristics

#### Methods

All the 12 studies finally selected for the review were randomized controlled trials published in English. Five studies were conducted in the United States, three in Italy, two in Spain, one in Canada, and one in China. Six studies (Borrego-Écija et al., 2023; Ferrucci et al., 2018; Hosseini et al., 2019; McConathey et al., 2017; Nissim et al., 2022; Roncero et al., 2017) adopted a crossover design (i.e., each participant underwent both active and sham intervention), three were parallel trials (Benussi et al., 2020; Cotelli et al., 2014; Huang et al., 2023), and two (de Aguiar et al., 2020a, 2020b; Wang et al., 2022) were first-phase reports of a prior crossover study (Tsapkini et al., 2018) and therefore considered as parallel studies; finally, one study (Pytel et al., 2021) reported a mixed design where some participants were assigned to the active condition and the other subsample, firstly allocated to the sham condition, then crossed to the active one (Table 1).

**Table 1:** Methodological characteristics of included studies

Study	Design	NiBS	SLT	Sessions	Stimulation parameters	Stimulation target	Linguistic test(s)	Post-treatment evaluation	Follow-up(s) evaluation(s)
Cotelli et al. (2014)	Parallel	tDCS	Picture-naming training	10 daily sessions(× 2 weeks)	5 × 5 ${\mathrm{cm}}^{2}$ electrodes; 2 mA, 20 min	Anode: 8 cm frontally and 6 cm laterally with respect to Cz Cathode: right arm	Picture naming, AAT^g^, grammar comprehension (BADA^h^), phonemic fluency, semantic fluency	Immediate post-treatment	12 weeks from baseline
McConathey et al. (2017)	Crossover	tDCS	None (narrative task only)	10 daily sessions (× 2 weeks)	5 × 5 ${\mathrm{cm}}^{2}$ electrodes, 1.5 mA, 20 min	Anode: F7 Cathode: O1	Sentence repetition, grammar comprehension (TROG^i^), semantic fluency, picture naming (BNT^j^), semantic associations (PPT^k^)	Immediate post-treatment	6 and 12 weeks post- treatment
Roncero et al. (2017)	Crossover	tDCS	Picture-naming training	10 daily sessions (× 3 weeks)	5 × 7 ${\mathrm{cm}}^{2}$ electrodes, 2 mA, 30 min	Anode: TP9 Cathode: right fronto-orbital area	Picture naming	Immediate post-treatment	2 weeks post-treatment
Ferrucci et al. (2018)	Crossover	tDCS	None	5 daily sessions (× 1 week)	5 × 7 ${\mathrm{cm}}^{2}$ electrodes, 2 mA, 20 min	Anode: F7 and F8 Cathode: right deltoid	Phonemic fluency, picture naming	Immediate post-treatment	1 and 4 weeks post-treatment
Hosseini et al. (2019)	Crossover	tDCS	None (narrative task only)	10 daily sessions (× 2 weeks)	5 × 5 ${\mathrm{cm}}^{2}$ electrodes, 1.5 mA, 20 min	Anode: F7 Cathode: O1	Semantic fluency, grammar comprehension (TROG), semantic associations (PPT), picture naming (BNT)	Immediate post-treatment	6 and 12 weeks post-treatment
Benussi et al. (2020)	Parallel	tDCS	None	10 daily sessions (× 2 weeks)	5 × 7 ${\mathrm{cm}}^{2}$ electrodes, 2 mA, 20 min	Anode: F3 Cathode: right deltoid	Phonemic fluency, semantic fluency	Immediate post-treatment	3 and 6 months from baseline
de Aguiar et al., (2020a, 2020b**)**	Parallel	tDCS	Picture-naming training	12 daily sessions on average (± 2) (× 3 weeks)	5 × 5 ${\mathrm{cm}}^{2}$ electrodes, 2 mA, 20 min	Anode: F7 Cathode: right cheek	Written picture naming	Immediate post-treatment	2 weeks and 2 months post-treatment
Pytel et al. (2021)	Mixed	rTMS	None	15 daily sessions (× 3 weeks)	20 Hz, 20-s inter-train, 1500 pulses, 100% RMT^a^	Personalized target: LIFG^b^ (9 patients), LSFG^c^ (3 patients), LDLPFC^d^ (6 patients), RSFG^e^ (1 patient), LATL^f^ (1 patient)	Story description, picture naming, story reading, repetition	Within the first 2 weeks post-treatment	None
Nissim et al. (2022)	Crossover	tDCS	Constraint-induced language therapy (CILT)	10 daily sessions (× 2 weeks)	1-cm diameter round electrodes, 1.5 mA, 20 min	Anode: FT7 Cathodes: F7, T7, FC5, FT9	Western aphasia battery-revised (WAB-R)	Immediate post-treatment	6 weeks post-treatment
Wang et al. (2022)	Parallel	tDCS	Picture-naming training	Approximately 12 daily sessions (× 3 weeks)	5 × 5 ${\mathrm{cm}}^{2}$ electrodes, 2 mA, 20 min	Anode: F7 Cathode: right cheek	Semantic and phonemic fluency	Immediate post-treatment	2 weeks and 2 months post-treatment
Borrego-Ecijia et al. (2023)	Crossover	tDCS	Unspecified SLT type	10 daily sessions (× 2 weeks)	1-cm radius round electrodes, max 4 mA, 26 min	Anodes: F7, FC1, FC5, P7 Cathodes: PO8, C1, FPZ	Phonemic fluency, semantic fluency, picture naming, single-word comprehension, semantic associations (Camel and Cactus test, PPT), reading	Immediate post-treatment	1 and 3 months post-treatment
Huang et al. (2023)	Parallel	rTMS	None	20 daily sessions (× 4 weeks)	10 Hz, 2-s inter-train, 1000 pulses, 120% RMT	LDLPFC	Picture naming, WAB	1-month post-treatment	3 and 6 months post-treatment

^a^*RMT* resting motor threshold

^b^*LIFG* left inferior frontal gyrus

^c^*LSFG* left superior frontal gyrus

^d^*LDLPFC* left dorsolateral prefrontal cortex

^e^*RSFG* right superior frontal gyrus

^f^*LATL* left anterior temporal lobe

^g^*AAT* Aachener Aphasie Test

^h^*BADA* battery for the analysis of the aphasic deficit

^i^*TROG* Penn-Test for reception of grammar

^j^*BNT* Boston Naming Test

^k^*PPT* Pyramids and Palm Trees Test

The included studies involved 229 formally diagnosed PPA patients overall (Table 2). Of these, 112 were diagnosed with the non-fluent/agrammatic variant, 53 with the semantic variant, and 59 with the logopenic variant; five patients from one study (Ferrucci et al., 2018) were not classified into a specific PPA subtype. The sample recruited in two studies (Benussi et al., 2020; Ferrucci et al., 2018) included both PPA and FTD-behavioral variant patients, so the respective authors were contacted to obtain experimental data from only PPA patients. The main exclusion criteria entailed the following: psychiatric disorders or neurological diseases other than PPA, any contraindication for tDCS or TMS, global cognitive decline (e.g., MMSE < 15), left-hand dominance, and non-native speakers.

**Table 2:** Main demographic and clinical characteristics of patients in included studies

Study	Sample size	PPA variant	Age (*M* ± SD)	Sex ratio (M/F)	Education years (*M* ± SD)	Years post-onset (*M* ± SD)	MMSE (*M* ± SD)
Cotelli et al. (2014)	16 (8 active, 8 sham)	16 nfvPPA	66.9 ± 8.2 (active: 63.4 ± 6.8, sham: 70.4 ± 6.8)	6/10 (active: 3/5, sham: 3/5)	8.2 ± 3.1 (active: 9.3 ± 3.2, sham: 7.1 ± 3.2)	Not specified	18.3 ± 4.5 (active: 18.0 ± 2.9, sham: 18.6 ± 2.9)
McConathey et al. (2017)	7	6 nfvPPA, 1 lvPPA	68.71 ± 6.97	2/5	13.86 ± 2.73	4.29 ± 1.89	24.40 ± 4.77
Roncero et al. (2017)	10	6 nfvPPA, 2 lvPPA, 2 svPPA	67.4 ± 6.26	7/3	14.9 ± 3.48	Not specified	18.2 ± 7.97
Ferrucci et al. (2018)	5	Not specified	68.8 ± 4.66	3/2	14 ± 4.18	Not specified	24.6 ± 3.05
Hosseini et al. (2019)	6	3 nfvPPA, 3 lvPPA	67 ± 10.6	2/4	14.3 ± 3.2	4.8 ± 1.6	26.8 ± 2.3
Benussi et al. (2020)	30 (19 active, 11 sham)	18 nfvPPA, 12 svPPA	Active: 62.72 ± 7.02, sham: 64.05 ± 8	Active: 62.72 ± 7.02, sham: 64.05 ± 8	Active: 12.11 ± 4.45, sham: 12.1 ± 3.02	Active: 2.97 ± 1.86, sham: 2.80 ± 1.98	Active: 18.63 ± 10.93, sham: 20.1 ± 8.48
de Aguiar et al., (2020a, 2020b)	40 (21 active, 19 sham) at pre- and post, 37 (19 active, 18 sham) at fu1, 36 (17 active, 19 sham) at fu2	15 nfvPPA, 8 svPPA, 17 lvPPA	67.68 ± 6.76 (active: 66.1 ± 7.7, sham: 69.4 ± 5.1)	22/18 (active: 12/9, sham: 10/9)	Not specified	4.75 ± 2.87	Not specified
Pytel et al. (2021)	20 (20 active, 7 sham)	14 nfvPPA, 6 svPPA	Active: 66.95 ± 7.24, sham: 66.14 ± 7.31	Active: 8/12, sham: 3/4	Active: 13.40 ± 4.38, sham: 13.86 ± 3.18	Active: 3.80 ± 3.90, sham: 3.71 ± 1.79	Not specified
Nissim et al. (2022)	11	2 nfvPPA, 2 svPPA, 7 lvPPA	66.55 ± 6.85 (tDCS: 66.29 ± 7.67, sham: 67 ± 6.16)	7/4 (tDCS: 4/3, sham: 3/1)	Not specified	3.64 ± 1.69 (tDCS: 4.14 ± 1.86, sham: 2.75 ± 0.96)	24.09 ± 2 0.63 (active: 23.14 ± 2.34, sham: 25.75 ± 2.5)
Wang et al. (2022)	36 (18 active, 18 sham)	13 nfvPPA, 9 svPPA, 14 lvPPA	Active: 66.17 ± 7.49, sham: 69.72 ± 5.42	19/17 (active: 9/9, sham: 10/8)	Not specified	Active: 5.17 ± 3.40, sham: 4.72 ± 2.55	Not specified
Borrego-Ecijia et al. (2023)	13	5 nfvPPA, 4 svPPA, 4 lvppa	64 ± 8.56	4/9	Not specified	4.1 ± 1.8	24.62 ± 3.1
Huang et al. (2023)	35 (18 active, 17 sham)	14 nfvPPA, 10 svPPA, 11 lvPPA	Not specified	16/19 (active: 8/10, sham: 8/9)	Not specified	Median (${\mathrm{IQR}}^{a}$) in months: 24.0 (9.0–36.0); active: 24.0 (6 – 24.0), sham (*M* ± *SD*): 38.9 ± 28.9	Median (*IQR*): 22.0 (21.0–24.0); active (*M* ± SD): 22.0 ± 2.0, sham (*M* ± SD) 23.1 ± 2.0

^a^*IQR* inter-quartile range

As shown in Table 1, most studies adopted tDCS as a neuromodulation method (Benussi et al., 2020; Borrego-Écija et al., 2023; Cotelli et al., 2014; de Aguiar et al., 2020a, 2020b; Ferrucci et al., 2018; Hosseini et al., 2019; McConathey et al., 2017; Nissim et al., 2022; Roncero et al., 2017; Wang et al., 2022), of which six performed some kind of SLT concomitant or in close proximity with tDCS intervention (Borrego-Écija et al., 2023; Cotelli et al., 2014; de Aguiar et al., 2020a, 2020b; Nissim et al., 2022; Roncero et al., 2017; Wang et al., 2022). The only two RCTs that adopted rTMS (Huang et al., 2023; Pytel et al., 2021) did not perform SLT. Targeted brain areas included left prefrontal and perisilvian regions, stimulated via excitatory protocols (anodal tDCS or high-frequency rTMS), except for the study by Ferrucci and colleagues (2018), that implemented a bilateral excitatory stimulation of frontal areas, and the work by Pytel and collaborators (2021), that opted for a personalized target approach. The stimulation focality greatly depends on the NiBS method adopted (traditional two-electrode tDCS, HD-tDCS, or rTMS).

The included studies administered a wide range of tasks to capture tDCS/rTMS effects on language functions (Table 1), but only three types of tasks (phonemic fluency, semantic fluency, and naming) fulfilled the established cut-off (≥ 5 studies) for the meta-analysis (see flow chart, Fig. 1). The research groups administered the linguistic tasks immediately after the treatment, except for two studies (Huang et al., 2023; Pytel et al., 2021) that opted for a more delayed post-treatment evaluation (1 month and within the first 2 weeks after treatment, respectively). Three studies (Cotelli et al., 2014; Nissim et al., 2022; Roncero et al., 2017) administered one follow-up language evaluation, and eight studies (Benussi et al., 2020; Borrego-Ecija et al., 2023; de Aguiar et al., 2020a, 2020b; Ferrucci et al., 2018; Hosseini et al., 2019; Huang et al., 2023; McConathey et al., 2017; Wang et al., 2022) included two follow-ups in their designs. One study (Pytel et al., 2021) did not include a follow-up evaluation. Moreover, some of the studies implementing SLT (Borrego-Écija et al., 2023; Cotelli et al., 2014; de Aguiar et al., 2020a, 2020b; Roncero et al., 2017) reported language scores both for trained items during SLT and untrained items.

Regarding the object naming task, in most cases, this was created ad hoc by the researchers by using sets of validated pictures (e.g., Boston Naming Test, Snodgrass and Vanderwart) balanced for psycholinguistic properties, and the total score was the number of correct answers (1 = correct answer, 0 = incorrect answer) (Borrego-Ecija et al., 2023; Cotelli et al., 2014; Ferrucci et al., 2018; Hosseini et al., 2019; McConathey et al., 2017; Roncero et al., 2017). The number of stimuli greatly varied between studies: from 15 in the study by McConathey et al. (2017) and Hosseini et al. (2019) to 96 in the study by Pytel et al. (2021). In one study (Nissim et al., 2022) the naming task was part of the Western Aphasia Battery-Revised (WAB-R) (Kertesz, 2006), a validated diagnostic tool for the assessment of language disturbances, with different scoring criteria: three points for correct naming, two points in case of a phonemic paraphasia, one point if the patient needed a tactile or phonemic cue to respond correctly. Cotelli et al. (2014) administered instead two naming tests: an experimental picture naming test and the naming subtest of the Aachener Aphasie Test (AAT) (De Bleser et al., 1986); since the latter includes also other stimuli categories besides objects (colors, pictured compounds nouns, pictured sentences), only the scores at the first test were considered for the meta-analysis.

Concerning fluency tests, some studies tested more (i.e., three) letters/categories at each time point (Benussi et al., 2020 for phonemic fluency; Borrego-Ecija et al., 2023; Hosseini et al., 2019; McConathey et al., 2017; Wang et al., 2022); hence, the score reflects the total number of words produced across the three letters/categories. Instead, for the other studies (Benussi et al., 2020 for semantic fluency; Ferrucci et al., 2018; Nissim et al., 2022), one letter/category was used.

All the studies included contributed with only one effect size per each analyzed outcome.

#### Outcomes

The following linguistic outcomes, reported by at least five studies, were analyzed:

- Object naming change score (post–pre variation) comparing the intervention (tDCS or rTMS) group change to control change
- Phonemic fluency change score comparing the intervention group to control
- Semantic fluency change score comparing the intervention group to control

For each outcome of interest, different analyses were carried out for the change from pre- to immediate post-treatment, pre-treatment to short-term follow-up (2–6 weeks after treatment), and pre-treatment to long-term follow-up (2–3 months after treatment). All these outcomes refer only to untrained items, i.e., items that were not directly addressed by the SLT (if present). Indeed, there were not enough studies (< 5) to perform the analyses on trained items.

Confrontation naming involves the retrieval of the label corresponding to the viewed stimulus (e.g., an object). Phonemic fluency is tested by asking the participant to produce the maximum number of words beginning with a given letter in 1 min, whereas semantic fluency is evaluated by asking the participant to produce the maximum number of words belonging to a given semantic category in one minute. Albeit these tasks share some lexical retrieval processes, they have partially distinct neural bases, as suggested by functional neuroimaging and studies with brain-injured individuals (Biesbroek et al., 2021). For this reason, we decided to maintain these outcomes separately.

The first assessment in each study took place in the first month after the end of the treatment: 10 of 12 studies performed the post-treatment evaluation immediately after the intervention, whereas 2 studies performed the assessment within 2 weeks (Pytel et al., 2021) or 1 month (Huang et al., 2023) after treatment. Follow-up evaluations ranged from 1 week (Ferrucci et al., 2018) to 6 months (Benussi et al., 2020; Huang et al., 2023) after treatment, with one or two follow-ups per study.

### Risk of Bias in Individual Studies

We used the RoB 2.0 tool to assess the risk of bias for each of the included studies. A summary of these assessments is provided in Table 3. The ratings for each signaling question of the scale are provided as supplementary materials (Table S2, S3). In terms of overall risk of bias, a high risk of bias was detected for most studies (9/12); two crossover trials (Ferrucci et al., 2018; Roncero et al., 2017) adopted a 2-month washout period that might have been not sufficient to avoid carryover effects, as suggested by Tsapkini and colleagues (2018); moreover, regarding the bias due to deviations from intended intervention, some studies made no explicit mention of medications or SLT outside the study. Notably, the “some concerns” judgment in the last domain refers to the absence, for the majority of studies, of a pre-specified analysis plan.

**Table 3:** The table displays, for each included study, the risk-of-bias judgment for each of the six domains of bias and for the overall risk of bias

Study	Bias arising from the randomization process	Bias arising from period and carryover effects (crossover trials only)	Bias due to deviations from intended interventions	Bias due to missing outcome data	Bias in the measurement of the outcome	Bias in the selection of the reported result	Overall risk of bias
Cotelli et al. (2014)	Some concerns	Not applicable	High	Low	Low	Some concerns	High
McConathey et al. (2017)	Some concerns	Low	High	High	Low	Some concerns	High
Roncero et al. (2017)	Some concerns	High	Low	Low	Low	Some concerns	High
Ferrucci et al. (2018)	Some concerns	High	Low	Low	Low	Some concerns	High
Hosseini et al. (2019)	Some concerns	Low	High	Low	Some concerns	Some concerns	High
Benussi et al. (2020)	Low	Not applicable	Low	Low	Low	Some concerns	Some concerns
de Aguiar et al., (2020a, 2020b)	Some concerns	Not applicable	Low	Low	Low	High	High
Pytel et al. (2021)	Some concerns	Not applicable	Low	Low	Low	Some concerns	Some concerns
Nissim et al. (2022)	Some concerns	Some concerns	High	Low	Some concerns	Some concerns	High
Wang et al. (2022)	Some concerns	Not applicable	Low	High	Low	High	High
Borrego-Ecija et al. (2023)	Some concerns	Low	High	High	Low	Some concerns	High
Huang et al. (2023)	Low	Not applicable	Low	Low	Low	Some concerns	Some concerns

### Results of Syntheses

Regarding the naming outcome, seven studies (Borrego-Ecijia et al., 2023; Ferrucci et al., 2018; Hosseini et al., 2019; Huang et al., 2023; McConathey et al., 2017; Pytel et al., 2021; Roncero et al., 2017) reported the mean and standard deviation of the number of correct answers, while the other two studies (Cotelli et al., 2014; de Aguiar et al., 2020a, 2020b) expressed the result as the percentage of correct answers. We decided to take the percentage as the measure of interest to make the studies more comparable, given that the number of items was different in each study; in addition, one study (de Aguiar et al., 2020a, 2020b), due to a different unit of analysis (letters instead of words) made not possible to retrieve the total number of items to transform the naming score. Finally, as stated previously, one study (Nissim et al., 2022) adopted a different scale of measurement for naming, but the transformation to SMD allowed a direct comparison to the other studies.

#### Object Naming—Immediate Post-treatment

Nine studies were included in the meta-analysis of immediate post data: six crossover studies (Borrego-Ecija et al., 2023; Ferrucci et al., 2018; Hosseini et al., 2019; McConathey et al., 2017; Nissim et al., 2022; Roncero et al., 2017), two parallel studies (Cotelli et al., 2014; de Aguiar et al., 2020a, 2020b) and one mixed study (Pytel et al., 2021). Data were obtained from the paper full text or supplementary materials (Cotelli et al., 2014; de Aguiar et al., 2020a, 2020b; Pytel et al., 2021; Roncero et al., 2017) or from correspondence with authors (Borrego-Ecija et al., 2023; Ferrucci et al., 2018; Hosseini et al., 2019; McConathey et al., 2017; Nissim et al., 2022).

The included RCTs enrolled 124 participants, after excluding one participant from the analysis due to a ceiling effect (i.e., maximum score at baseline maintained throughout the study) (McConathey et al., 2017), and three participants due to missing data (Hosseini et al., 2019; Nissim et al., 2022). Eight of the trials were judged at high risk of bias (Borrego-Ecija et al., 2023; Cotelli et al., 2014; de Aguiar et al., 2020a, 2020b; Ferrucci et al., 2018; Hosseini et al., 2019; McConathey et al., 2017; Nissim et al., 2022; Roncero et al., 2017) whereas one showed only some concerns (Pytel et al., 2021).

**Fig. 2:**
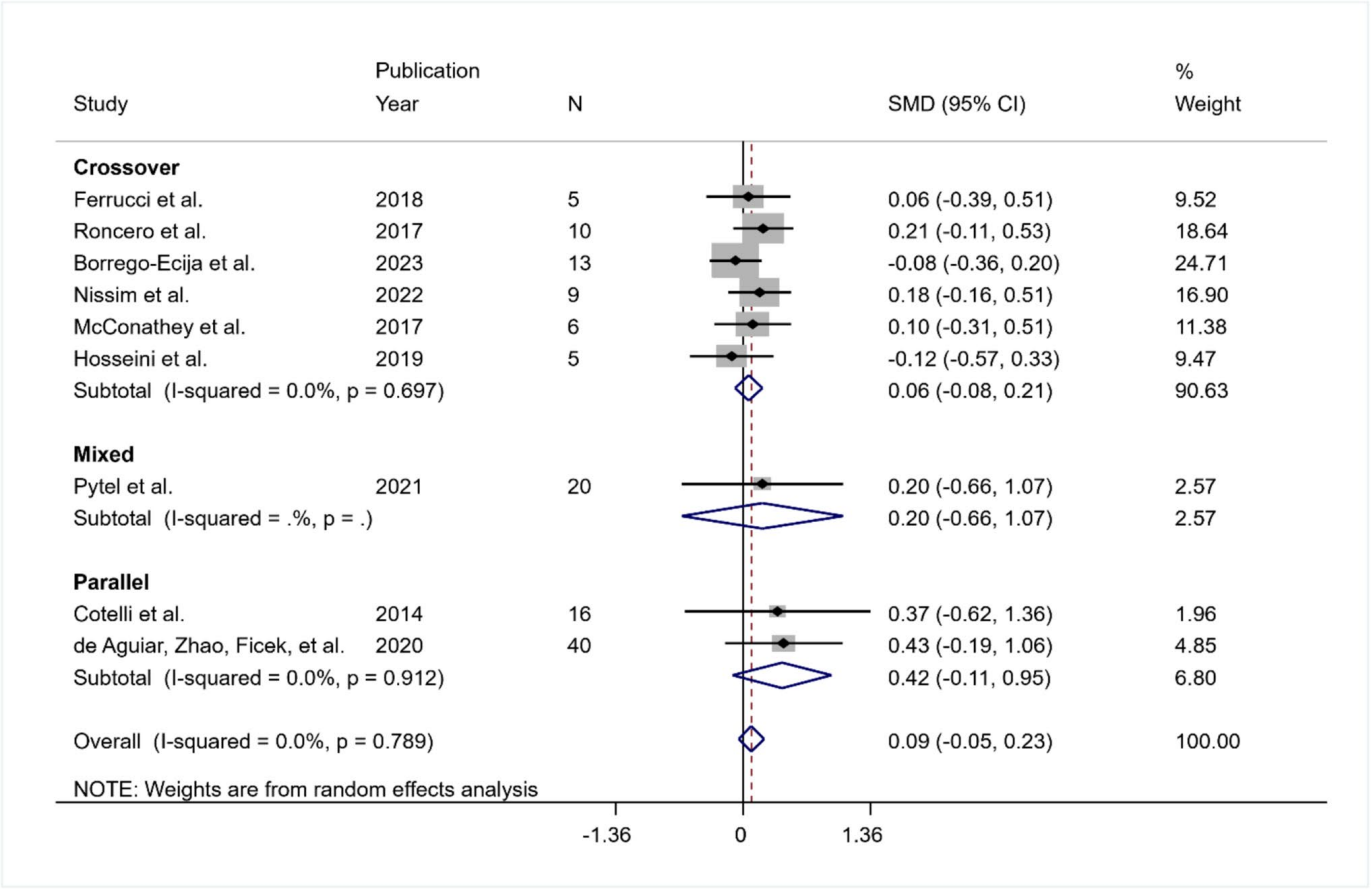
Summary results of the meta-analysis regarding naming differences between the active and sham conditions at post-treatment. The figure reports the SMD, 95% confidence interval (CI), and relative weight of each study. A positive (negative) effect size indicates a better performance in the active (sham) condition. Individual studies are clustered into three subgroups according to the study design (crossover, mixed, parallel). Combined effect sizes, with their respective confidence interval, are reported for the overall analysis and for each subgroup

The pooled SMD was equal to 0.09 indicating a major increment post real intervention than post sham, but not significant (95% CI − 0.05 to 0.23; *p* = 0.206). The heterogeneity was not significant (${I}^{2}$ = 0%; *Q* (df = 8) = 4.70, *p* = 0.789; tau-squared = 0.0). The pooled SMD was equal to 0.06 (95% CI − 0.08 to 0.21; *p* = 0.407) in the crossover group and equal to 0.42 (95% CI − 0.11 to 0.95; *p* = 0.124) in the parallel group. In both groups, the heterogeneity was not significant (${I}^{2}$ = 0% and tau-squared = 0.0 in both groups; in the crossover group *Q* (df = 5) = 3.02, *p* = 0.697 and in the parallel group *Q* (df = 1) = 0.01, *p* = 0.912) (Fig. 2).

##### Sensitivity Analysis

Considering only the studies performing language training, a sensitivity analysis was performed. Five studies were included in the analysis (Borrego-Ecija et al., 2023; Cotelli et al., 2014; de Aguiar et al., 2020a, 2020b; Nissim et al., 2022; Roncero et al., 2017), with a pooled SMD equal to 0.13 (95% CI − 0.06 to 0.31; *p* = 0.186; ${I}^{2}$ = 13.3%; *Q* (df = 4) = 3.66,* p* = 0.454; tau-squared = 0.01*)*.

##### Subgroup Analysis

**Fig. 3:**
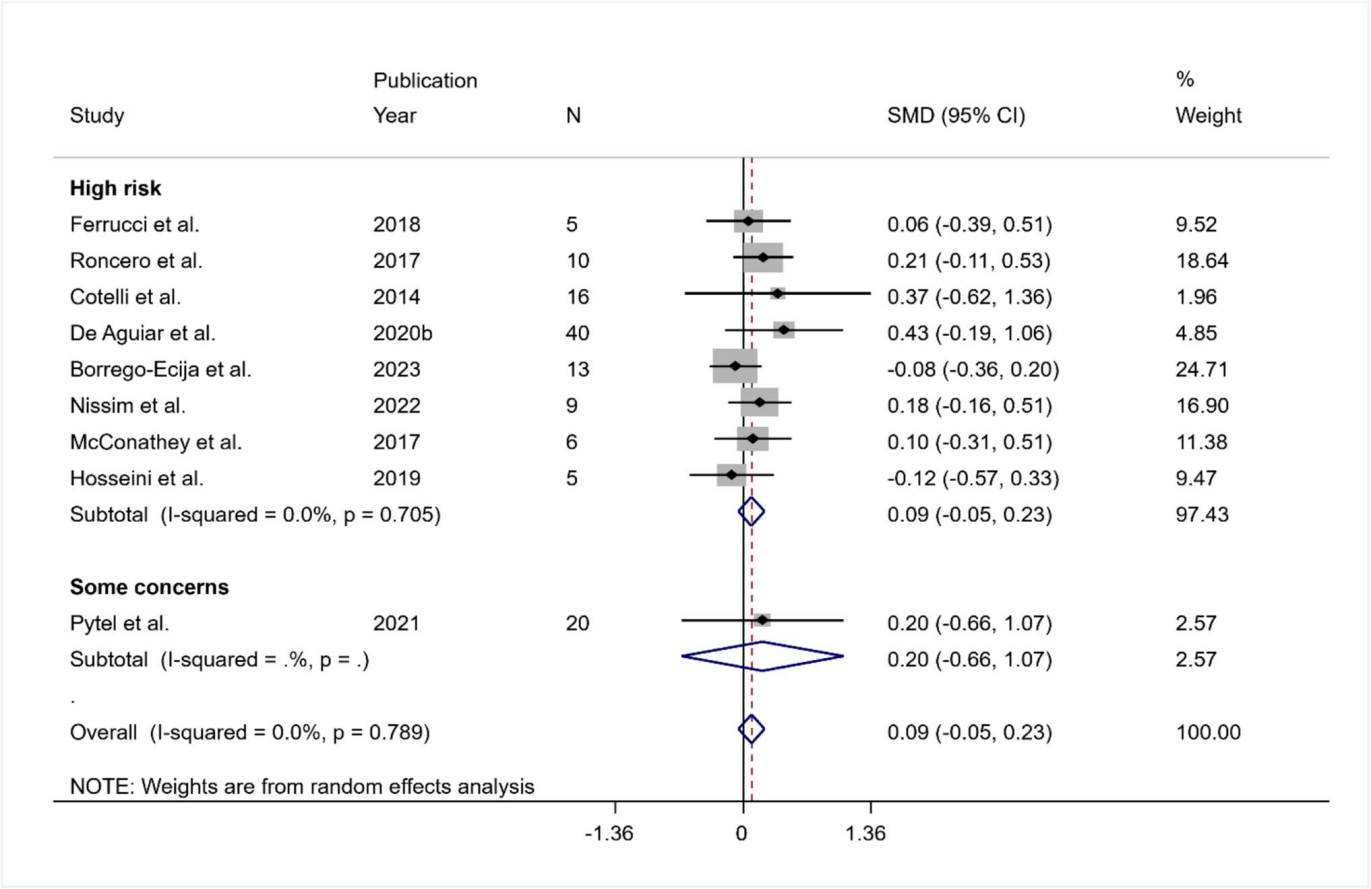
Summary results on naming (post-treatment) according to the risk of bias of each study. The figure reports the SMD, 95% confidence interval (CI), and relative weight of each study. A positive (negative) effect size indicates a better performance in the active (sham) condition. Individual studies are clustered into two subgroups according to the risk of bias evaluated with RoB-2 (high risk, some concern). Combined effect sizes, with their respective confidence interval, are reported for the overall analysis and for both subgroups

Given the low number of studies, the meta-regression approach could not be applied. From a descriptive point of view, the 8 studies with high risk obtained a non-significant pooled SMD equal to 0.086 (95% CI − 0.05 to 0.23), and the one study with some concerns resulted in a pooled SMD equal to 0.20 (95% CI − 0.66 to 1.07). The heterogeneity was not significant in the high-risk subgroup (${I}^{2}$ = 0%; tau-squared = 0; *Q* (df = 7) = 4.63, *p* = 0.705) (Fig. 3).

#### Object Naming—Short-Term Follow-Up

Eight studies entered the analysis of data observed from 2 to 6 weeks after treatment: six crossover studies (Borrego-Ecija et al., 2023; Ferrucci et al., 2018; Hosseini et al., 2019; McConathey et al., 2017; Nissim et al., 2022; Roncero et al., 2017) and two parallel studies (de Aguiar et al., 2020a, 2020b; Huang et al., 2023). Evaluations were carried out at 1 week (Ferrucci et al., 2018), 2 weeks (de Aguiar et al., 2020a, 2020b; Roncero et al., 2017), 4 weeks (Borrego-Ecija et al., 2023; Huang et al., 2023), and 6 weeks (Hosseini et al., 2019; McConathey et al., 2017; Nissim et al., 2022) after the treatment. Data were obtained from the paper full text (de Aguiar et al., 2020a, 2020b; Huang et al., 2023; Roncero et al., 2017) or correspondence with authors (Borrego-Ecija et al., 2023; Ferrucci et al., 2018; Hosseini et al., 2019; McConathey et al., 2017; Nissim et al., 2022).

The included RCTs enrolled 119 participants, after excluding one participant from the analysis due to a ceiling effect (i.e., maximum score at baseline maintained throughout the study) (McConathey et al., 2017), four participants due to missing data (Hosseini et al., 2019; Nissim et al., 2022), and three participants because of drop out (de Aguiar et al., 2020a, 2020b). Seven of the eight trials were judged at high risk of bias (Borrego-Ecija et al., 2023; Cotelli et al., 2014; de Aguiar et al., 2020a, 2020b; Ferrucci et al., 2018; Hosseini et al., 2019; McConathey et al., 2017; Nissim et al., 2022; Roncero et al., 2017), with one showing some concerns (Huang et al., 2023).

The pooled SMD was equal to 0.04, indicating a very small effect of treatment that was not significant (95% CI − 0.14 to 0.23; *p* = 0.658). The heterogeneity was moderate and significant (${I}^{2}$ = 53.5%; *Q* (df = 7) = 15.04, *p* = 0.036; tau-squared = 0.035).

**Fig. 4:**
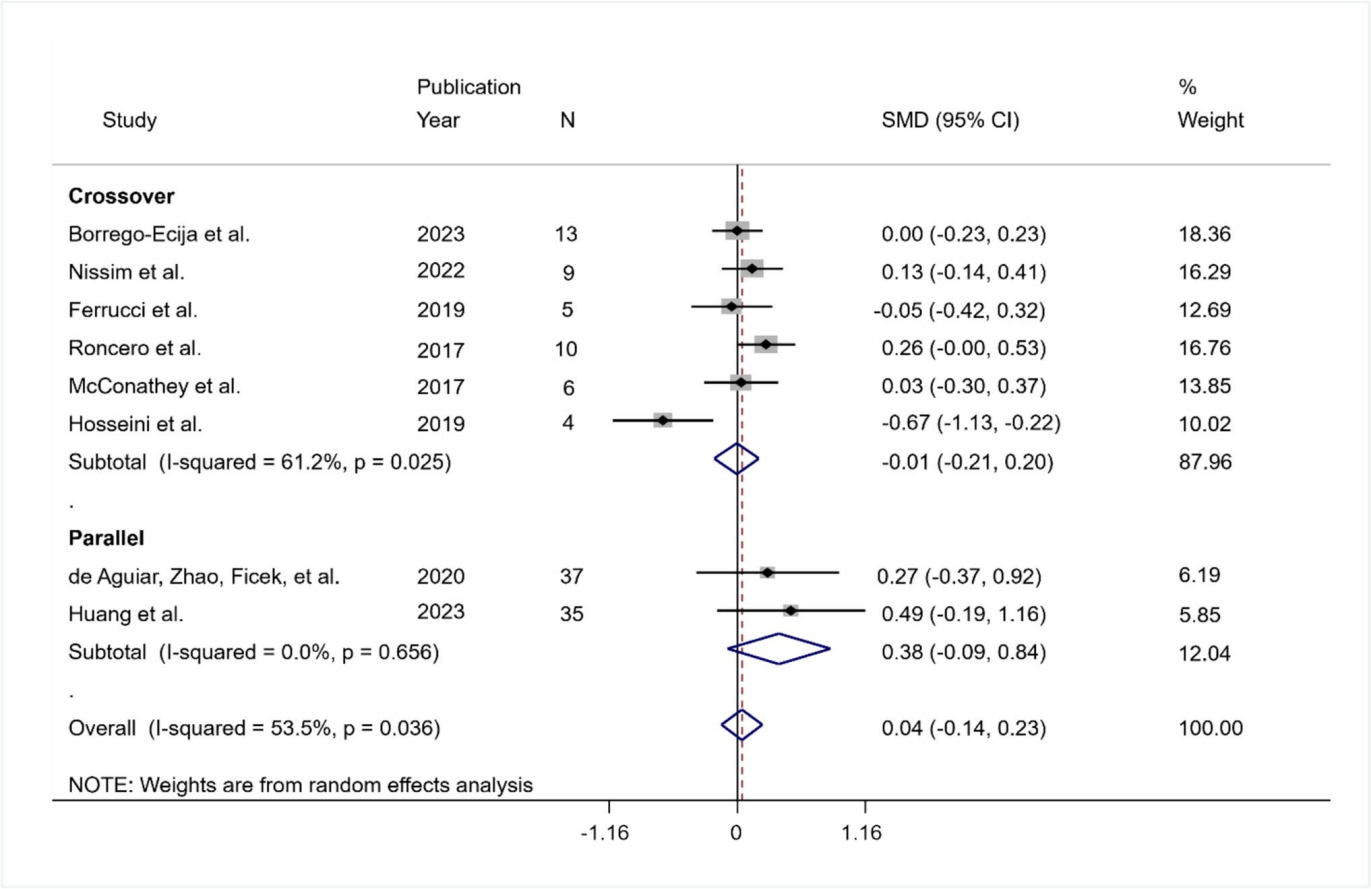
Summary results of the meta-analysis regarding naming differences between the active and sham conditions at short-term follow-up. The figure reports the SMD, 95% confidence interval (CI), and relative weight of each study. A positive (negative) effect size indicates a better performance in the active (sham) condition. Individual studies are clustered into three subgroups according to the study design (crossover, mixed, parallel). Combined effect sizes, with their respective confidence interval, are reported for the overall analysis and for each subgroup

The pooled SMD was equal to − 0.01 (95% CI − 0.21 to 0.20; *p* = 0.956) in the crossover group and equal to 0.38 (95% CI − 0.09 to 0.84; *p* = 0.113) in the parallel group. The heterogeneity was significant in the crossover group (${I}^{2}$ = 61.2%, *Q* (df = 5) = 12.88, *p* = 0.025; tau-squared = 0.038) but not in parallel group (${I}^{2}$ = 0%, *Q* (df = 1) = 0.2, *p* = 0.656; tau-squared = 0.00) (Fig. 4).

##### Sensitivity Analysis

The sensitivity analysis, considering only the studies performing language training (Borrego-Ecija et al., 2023; De Aguiar et al., 2020b; Nissim et al., 2022; Roncero et al., 2017), yielded a pooled SMD equal to 0.13 (95% CI − 0.02 to 0.27; *p* = 0.085; ${I}^{2}$ = 0%; *Q* (df = 3) = 2.37, *p* = 0.500; tau-squared = 0.00).

##### Subgroup Analysis

The studies at high risk of bias obtained a non-significant pooled SMD equal to 0.015 (95% CI − 0.17 to 0.203), while the only study with “some concerns” judgment had a pooled *SMD* equal to 0.49 (95% CI − 0.19 to 1.16). The heterogeneity was significant in the high-risk subgroup (${I}^{2}$ = 55.2%; *Q* (df = 6) = 13.4, *p* = 0.037; tau-squared = 0.034).

#### Object Naming—Long-Term Follow-Up

Six studies were included in the meta-analysis of long-term follow-up data (i.e., from 2 to 3 months after treatment): three crossover studies (Borrego-Ecija et al., 2023; Hosseini et al., 2019; McConathey et al., 2017) and three parallel studies (Cotelli et al., 2014; de Aguiar et al., 2020a, 2020b; Huang et al., 2023). Data were obtained from the paper’s full text or supplementary materials (Cotelli et al., 2014; de Aguiar et al., 2020a, 2020b; Huang et al., 2023) or from correspondence with authors (Borrego-Ecija et al., 2023; Hosseini et al., 2019; McConathey et al., 2017). Assessments were conducted at 2 months (de Aguiar et al., 2020a, 2020b), 10 weeks (Cotelli et al., 2014), and 3 months (Borrego-Ecija et al., 2023; Hosseini et al., 2019; Huang et al., 2023; McConathey et al., 2017) after the end of the treatment.

The included RCTs enrolled 111 participants. In the study by de Aguiar et al., (2020a, 2020b), four participants dropped out at the second follow-up, leading to a final sample of 36 participants. One additional participant was excluded due to a ceiling effect (i.e., maximum score at baseline maintained throughout the study) (McConathey et al., 2017), and one participant due to missing data (Hosseini et al., 2019). Risk of bias assessment led to a high-risk judgment for all studies except for the one by Huang et al. (2023).

The pooled SMD was equal to 0.06 indicating a small effect of intervention in naming but not significant (95% CI − 0.3 to 0.41; *p* = 0.775). The heterogeneity was significant (${I}^{2}$ = 71.8%; *Q* (df = 5) = 17.7, *p* = 0.003; tau-squared = 0.122).

**Fig. 5:**
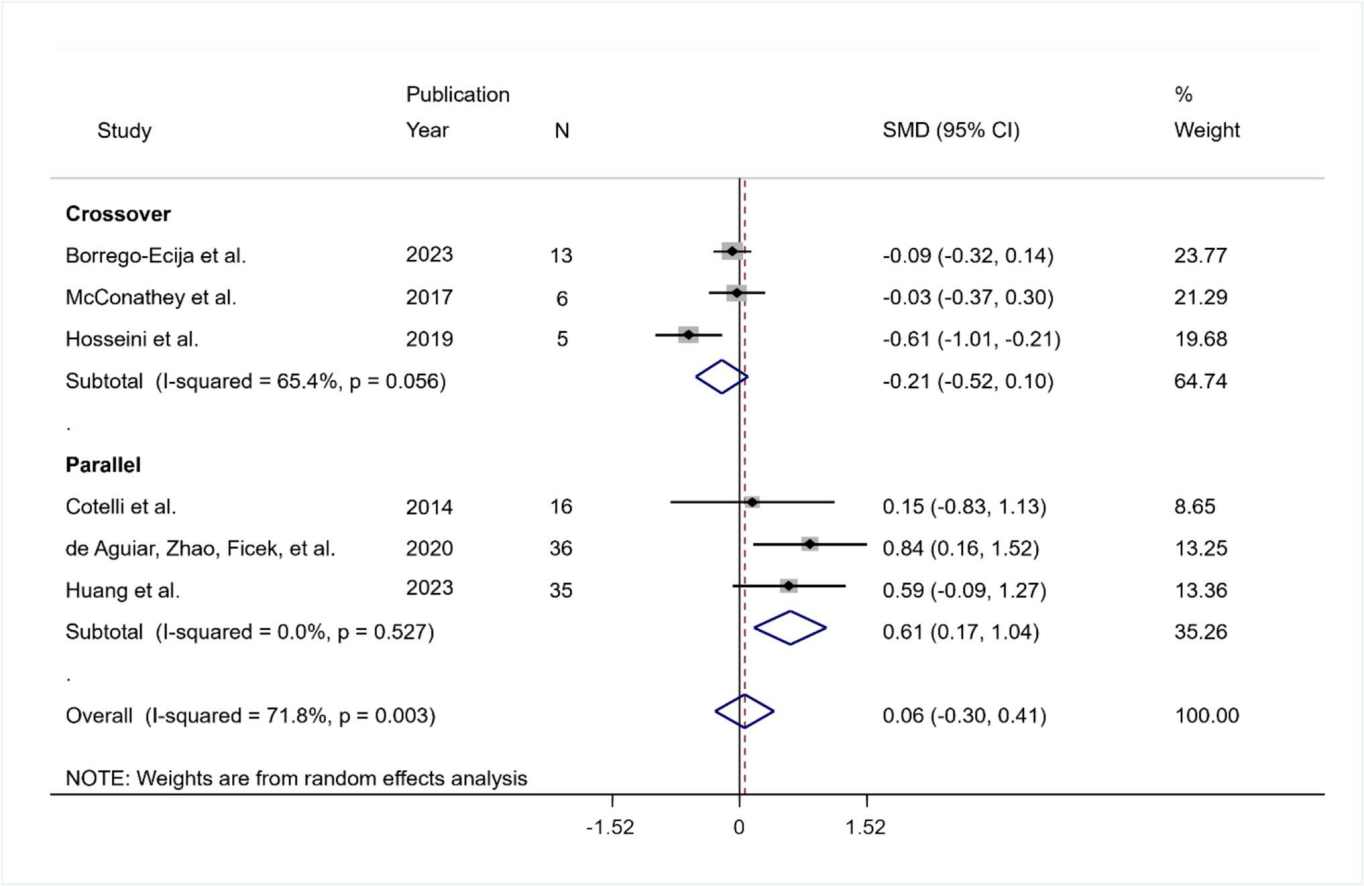
Summary results of the meta-analysis regarding naming differences between the active and sham conditions at long-term follow-up. The figure reports the SMD, 95% confidence interval (CI), and relative weight of each study. A positive (negative) effect size indicates a better performance in the active (sham) condition. Individual studies are clustered into three subgroups according to the study design (crossover, mixed, parallel). Combined effect sizes, with their respective confidence interval, are reported for the overall analysis and for each subgroup

The pooled SMD was equal to − 0.21 indicating a lower effect of the real intervention compared to sham but non-significant (95% CI − 0.52 to 0.10; *p* = 0.178) in the crossover group and equal to 0.61 indicating a significant moderate effect of the intervention (95% CI 0.17 to 1.04; *p* = 0.006) in the parallel group. In both groups, the heterogeneity was not significant (in the crossover group ${I}^{2}$ = 65.4%, *Q* (df = 2) = 5.78, *p* = 0.056, tau-squared = 0.049; in the parallel group ${I}^{2}$ = 0%, *Q* (df = 2) = 1.28, *p* = 0.527, tau-squared = 0.00) (Fig. 5).

##### Sensitivity Analysis

The sensitivity analysis, performed on the five studies that included language training in their design (Borrego-Ecija et al., 2023; Cotelli et al., 2014; de Aguiar et al., 2020a, 2020b; Hosseini et al., 2019; McConathey et al., 2017), resulted in a pooled SMD equal to 0.20 (95% CI − 0.25 to 0.65; *p* = 0.374; ${I}^{2}$ = 38.6%; *Q* (df = 4) = 6.52, *p* = 0.164; tau-squared = 0.095).

##### Subgroup Analysis

Five studies with high risk obtained a non-significant pooled SMD equal to − 0.032 (95% CI − 0.39 to 0.33), and only one study with some concerns pooled SMD = 0.59 (95% CI − 0.09 to 1.26). The heterogeneity was significant in high-risk subgroups (${I}^{2}$ = 71.3%; *Q* (df = 4) = 13.9, *p* = 0.007; tau-squared = 0.106).

#### Phonemic Fluency—Immediately Post-treatment

Regarding phonemic fluency, five studies, three parallel (Benussi et al., 2020; Cotelli et al., 2014; Wang et al., 2022), and two crossovers (Borrego-Ecija et al., 2023; Ferrucci et al., 2018) were included in the meta-analysis of immediate post-treatment data, for a total amount of 100 participants. Data were obtained from the paper’s full text or supplementary materials (Wang et al., 2022) or from correspondence with authors (Benussi et al., 2020; Borrego-Ecija et al., 2023; Ferrucci et al., 2018).

The pooled SMD was equal to − 0.07 indicating that the mean change in the control group was larger than the mean variation in the intervention group but not significant (95% CI − 0.28 to 0.15; *p* = 0.536). The heterogeneity was not significant (${I}^{2}$ = 0%; *Q* (df = 4) = 2.12, *p* = 0.714; tau-squared = 0.00).

**Fig. 6:**
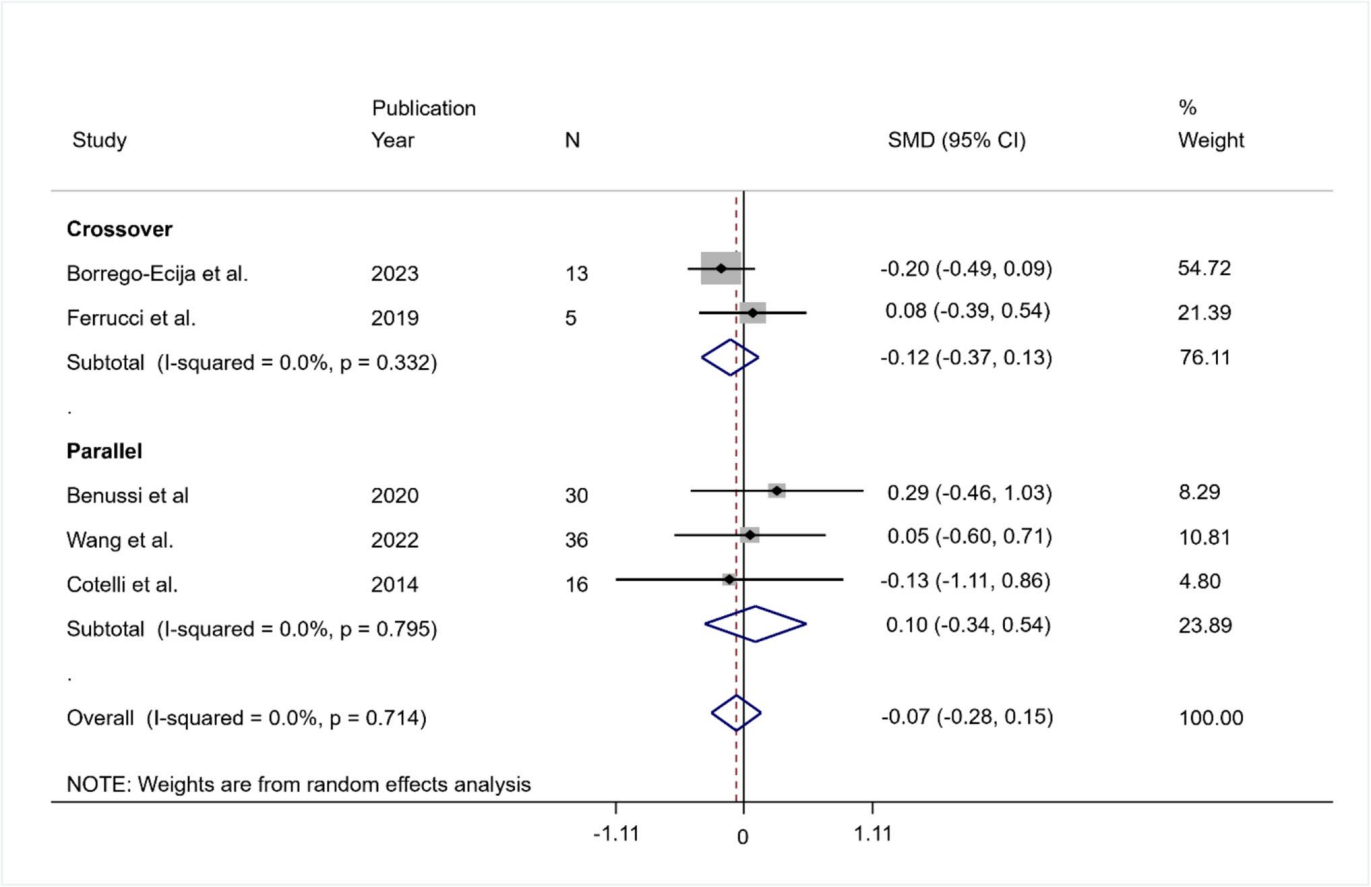
Summary results of the meta-analysis regarding phonemic fluency differences between the active and sham conditions at post-treatment. The figure reports the SMD, 95% confidence interval (CI), and relative weight of each study. A positive (negative) effect size indicates a better performance in the active (sham) condition. Individual studies are clustered into three subgroups according to the study design (crossover, mixed, parallel). Combined effect sizes, with their respective confidence interval, are reported for the overall analysis and for each subgroup

The pooled SMD was equal to − 0.12 (95% CI − 0.37 to 0.13; *p* = 0.340) in the crossover group and equal to 0.10 (95% CI − 0.34 to 0.54; *p* = 0.663) in the parallel group. In both groups, the heterogeneity was not significant (in crossover group ${I}^{2}$ = 0%, *Q* (df = 1) = 0.94, *p* = 0.332, tau-squared = 0.00; in parallel group ${I}^{2}$ = 0.0%, *Q* (df = 2) = 0.46, *p* = 0.795, tau-squared = 0.00) (Fig. 6).

##### Subgroup Analysis

Considering the RoB-2 score, all studies were at high risk except for the study by Benussi et al. (2020), which had some concerns.

##### Sensitivity Analysis

Among the studies, only Borrego-Ecijia et al. (2023) performed training.

#### Phonemic Fluency—Short-Term Follow-Up

One parallel (Wang et al., 2022) and two crossover studies (Borrego-Ecija et al., 2023; Ferrucci et al., 2018) were included in the meta-analysis of short-term follow-up data (2–6 weeks). Because of the subthreshold number of studies (< 5), the statistical synthesis was not undertaken.

#### Phonemic Fluency—Long-Term Follow-Up

Three parallel (Benussi et al., 2020; Cotelli et al., 2014; Wang et al., 2022) and one crossover study (Borrego-Ecija et al., 2023) were included in the meta-analysis of short-term follow-up data (2–3 months). Because of the subthreshold number of studies (< 5), the statistical synthesis was not conducted.

#### Semantic Fluency—Immediate Post-treatment

Seven studies, four crossovers (Borrego-Ecija et al., 2023; Hosseini et al., 2019; McConathey et al., 2017; Nissim et al., 2022), and three parallel studies (Benussi et al., 2020; Cotelli et al., 2014; Wang et al., 2022) were included. Data were obtained from the paper’s full text or supplementary materials (Wang et al., 2022) or from correspondence with authors (Benussi et al., 2020; Borrego-Ecija et al., 2023; Hosseini et al., 2019; McConathey et al., 2017; Nissim et al., 2022). The data from 117 participants in total were meta-analyzed after two participants were excluded from the original samples due to missing data (Nissim et al., 2022). Data were obtained from the paper’s full text or supplementary materials (Wang et al., 2022) or from correspondence with authors (Benussi et al., 2020; Borrego-Ecija et al., 2023; Hosseini et al., 2019; McConathey et al., 2017; Nissim et al., 2022).

The pooled SMD was equal to 0.06, indicating that the mean change in control was larger than the mean variation in the intervention group but not significant (95% CI − 0.08 to 0.20; *p* = 0.379). Also, the heterogeneity was not significant (${I}^{2}$= 0%; *Q* (df = 6) = 5.77, *p* = 0.450; tau-squared = 0.00).

**Fig. 7:**
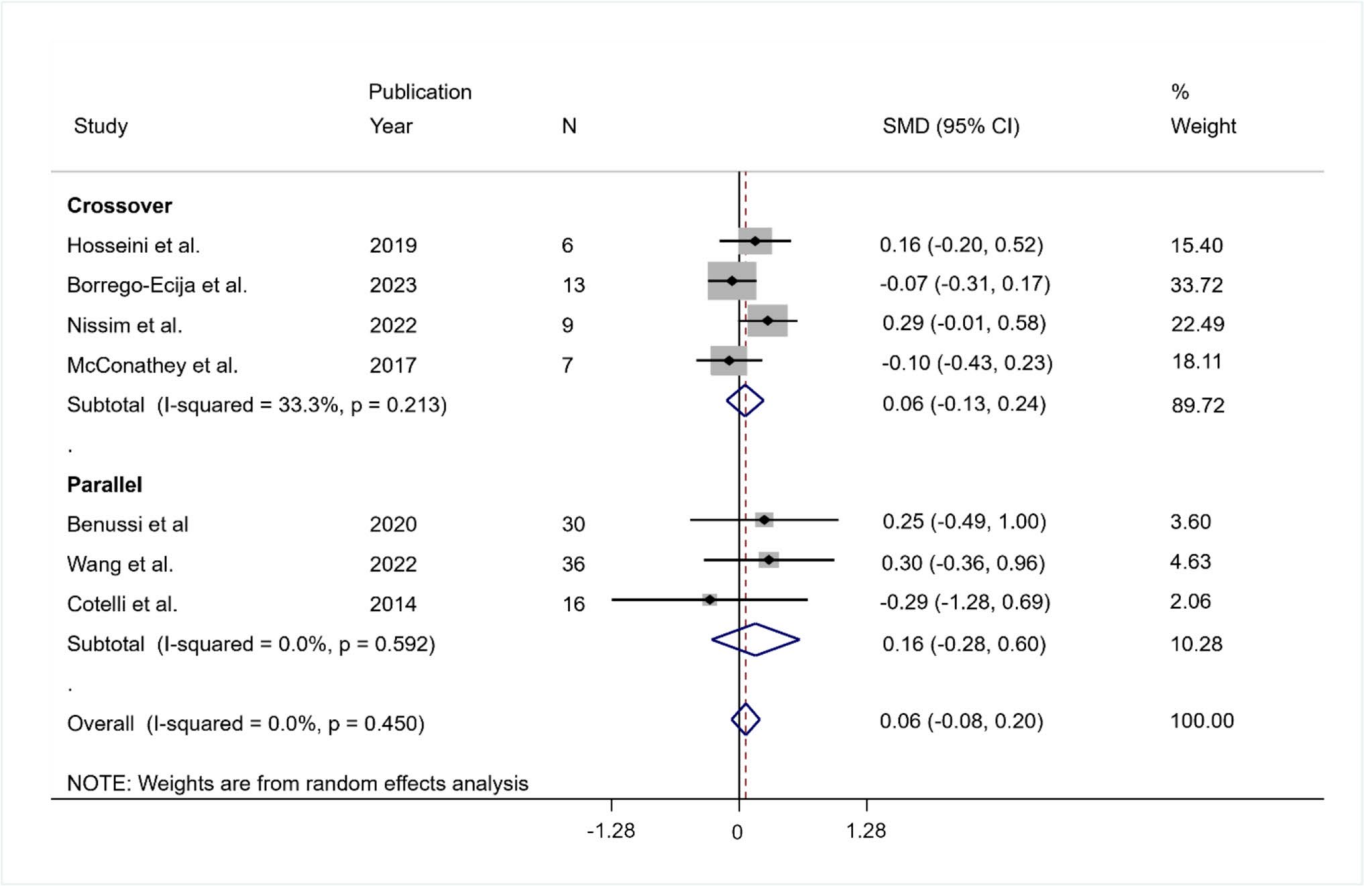
Summary results of the meta-analysis regarding semantic fluency differences between the active and sham conditions at post-treatment. The figure reports the SMD, 95% confidence interval (CI), and relative weight of each study. A positive (negative) effect size indicates a better performance in the active (sham) condition. Individual studies are clustered into three subgroups according to the study design (crossover, mixed, parallel). Combined effect sizes, with their respective confidence interval, are reported for the overall analysis and for each subgroup

The pooled effect size was equal to 0.06 (95% CI − 0.13 to 0.24; *p* = 0.534) in the crossover group (4 studies) and equal to 0.16 (95% CI − 0.28 to 0.60; *p* = 0.467) in the parallel group (3 studies). In both groups, the heterogeneity was not significant (in the crossover group: ${I}^{2}$= 33.3%, *Q* (df = 3) = 4.5, *p* = 0.213, tau-squared = 0.012; in the parallel group ${I}^{2}$= 0.0%, *Q* (df = 2) = 1.05, *p* = 0.592, tau-squared = 0.00) (Fig. 7).

##### Subgroup Analysis

Considering the RoB-2 score, all studies were at high risk except for the study by Benussi et al. (2020), which showed some concerns.

##### Sensitivity Analysis

Among the included studies, only Borrego-Ecijia et al. (2023), Cotelli et al. (2014), and Nissim et al. (2022) performed training. The pooled SMD was equal to − 0.03 (95% CI − 0.44 to 0.39; *p* = 0.902); the heterogeneity was not significant (${I}^{2}$ = 0%, *Q* (df = 2) = 0.82, *p* = 0.663, tau-squared = 0.00).

#### Semantic Fluency—Short-Term Follow-Up

Four crossover studies (Borrego-Ecija et al., 2023, Hosseini et al., 2019; McConathey et al., 2017; Nissim et al., 2022) and one parallel study (Wang et al., 2022) were included in the meta-analysis of short-term follow-up data (2–6 weeks) on semantic fluency. The data from 69 participants in total were meta-analyzed after two participants were excluded from the original samples due to missing data (Nissim et al., 2022) and two participants dropped out (Wang et al., 2022). Data were obtained from the paper’s full text or supplementary materials (Wang et al., 2022) or from correspondence with authors (Borrego-Ecija et al., 2023; Hosseini et al., 2019; McConathey et al., 2017; Nissim et al., 2022).

**Fig. 8:**
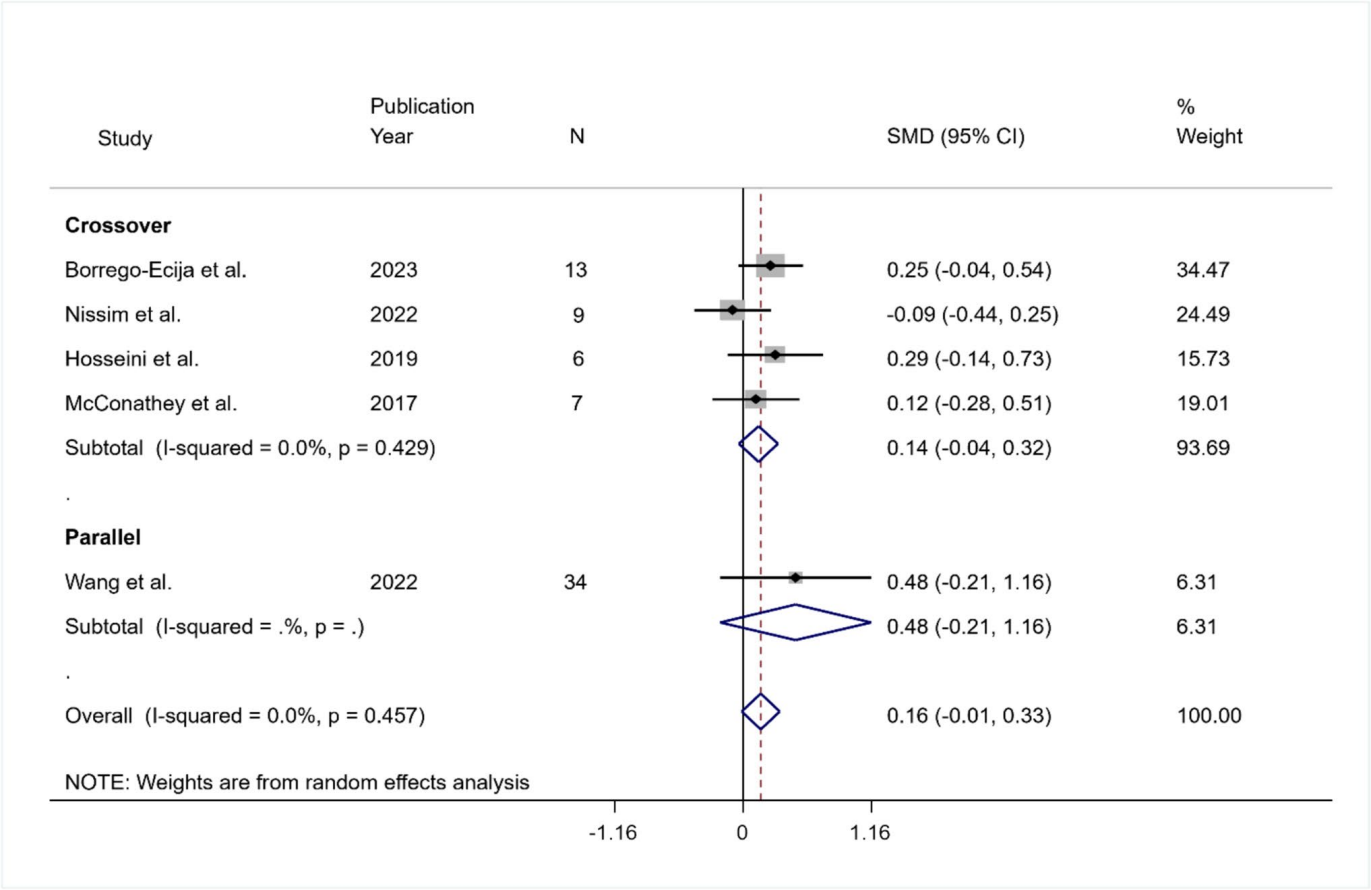
Summary results of the meta-analysis regarding semantic fluency differences between the active and sham conditions at short-term follow-up. The figure reports the SMD, 95% confidence interval (CI), and relative weight of each study. A positive (negative) effect size indicates a better performance in the active (sham) condition. Individual studies are clustered into three subgroups according to the study design (crossover, mixed, parallel). Combined effect sizes, with their respective confidence interval, are reported for the overall analysis and for each subgroup

The pooled SMD was equal to 0.16 but was not significant (95% CI − 0.01 to 0.33; *p* = 0.066). The heterogeneity was not significant (${I}^{2}$ = 0%; *p* = 0.457). The pooled SMD was equal to 0.14 (95% CI − 0.04 to 0.32; *p* = 0.122) in the crossover group, and the heterogeneity was not significant (${I}^{2}$ = 0%, *Q* (df = 4) = 3.64, *p* = 0.429; tau-squared = 0.00) (Fig. 8).

##### Subgroup Analysis

Considering the risk-of-bias assessment, all the studies were judged at high risk.

##### Sensitivity Analysis

Among the studies, only Borrego-Ecijia et al. (2023) and Nissim et al. (2022) performed training. The respective estimated effects are contrasting although not significant: SMD = 0.25 (95% CI: − 0.04 to 0.54) and SMD = − 0.09 (95% CI − 0.44 to 0.25), respectively.

#### Semantic Fluency—Long-Term Follow-Up

Three crossover studies (Borrego-Ecija et al., 2023; Hosseini et al., 2019; McConathey et al., 2017) and three parallel studies (Benussi et al., 2020; Cotelli et al., 2014; Wang et al., 2022) were included in this analysis of long-term follow-up data (2–3 months) on semantic fluency. The data from 102 participants in total were meta-analyzed after six participants were excluded from the original samples due to dropout (Wang et al., 2022). Data were obtained from the paper’s full text or supplementary materials (Wang et al., 2022) or from correspondence with authors (Benussi et al., 2020; Borrego-Ecija et al., 2023; Cotelli et al., 2014; Hosseini et al., 2019; McConathey et al., 2017).

**Fig. 9:**
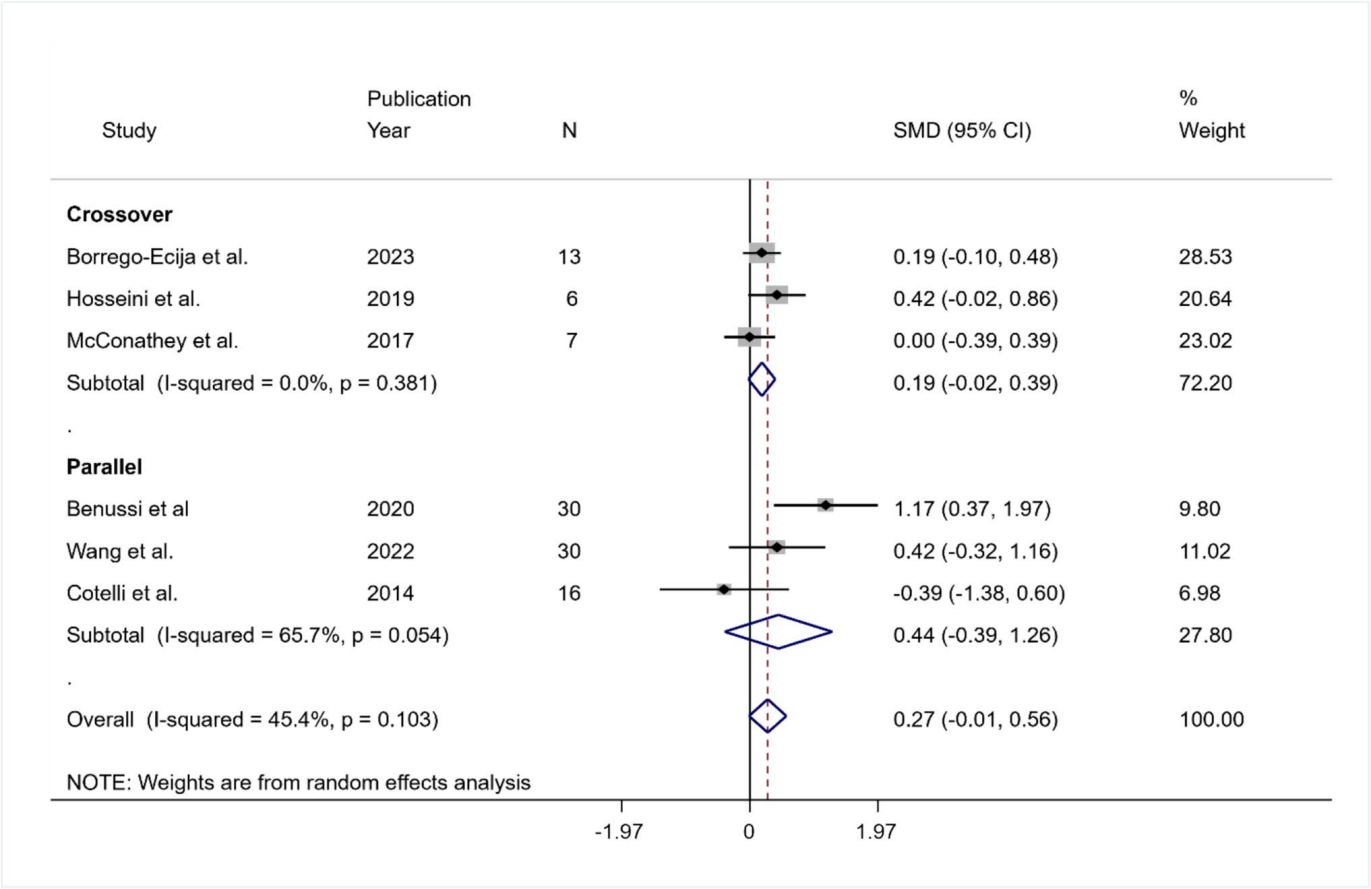
Summary results of the meta-analysis regarding semantic fluency differences between the active and sham conditions at long-term follow-up. The figure reports the SMD, 95% confidence interval (CI), and relative weight of each study. A positive (negative) effect size indicates a better performance in the active (sham) condition. Individual studies are clustered into three subgroups according to the study design (crossover, mixed, parallel). Combined effect sizes, with their respective confidence interval, are reported for the overall analysis and for each subgroup

The pooled SMD was equal to 0.27 but was not significant (95% CI − 0.01 to 0.56; *p* = 0.063). The heterogeneity was not significant (${I}^{2}$ = 45.4%; *p* = 0.103). In the crossover group, the pooled *SMD* was equal to 0.19 (95% CI: − 0.02 to 0.33; *p* = 0.077), and the heterogeneity was not significant (${I}^{2}$ = 0%, *p* = 0.381); in the parallel group, the SMD was equal to 0.44 (95% CI − 0.39 to 1.26; *p* = 0.297), and the heterogeneity was moderate and borderline significant (${I}^{2}$ = 5.7%, *Q* (df = 5) = 9.15, *p* = 0.05; tau-squared = 0.053) due to the difference in the direction of the estimated effect sizes for Benussi et al. (2020) and Cotelli et al. (2014) (Fig. 9).

##### Subgroup Analysis

Considering the RoB-2, all studies were at high risk, with the exception of Benussi et al. (2020).

##### Sensitivity Analysis

Among the studies, only Borrego-Ecijia et al. (2023) and Cotelli et al. (2014) performed training. The respective SMDs show different directions of effect, although not significant: SMD = 0.19 (95% CI − 0.10 to 0.48) and SMD = − 0.39 (95% CI − 1.38 to 0.60), respectively.

### Reporting Biases

**Fig. 10:**
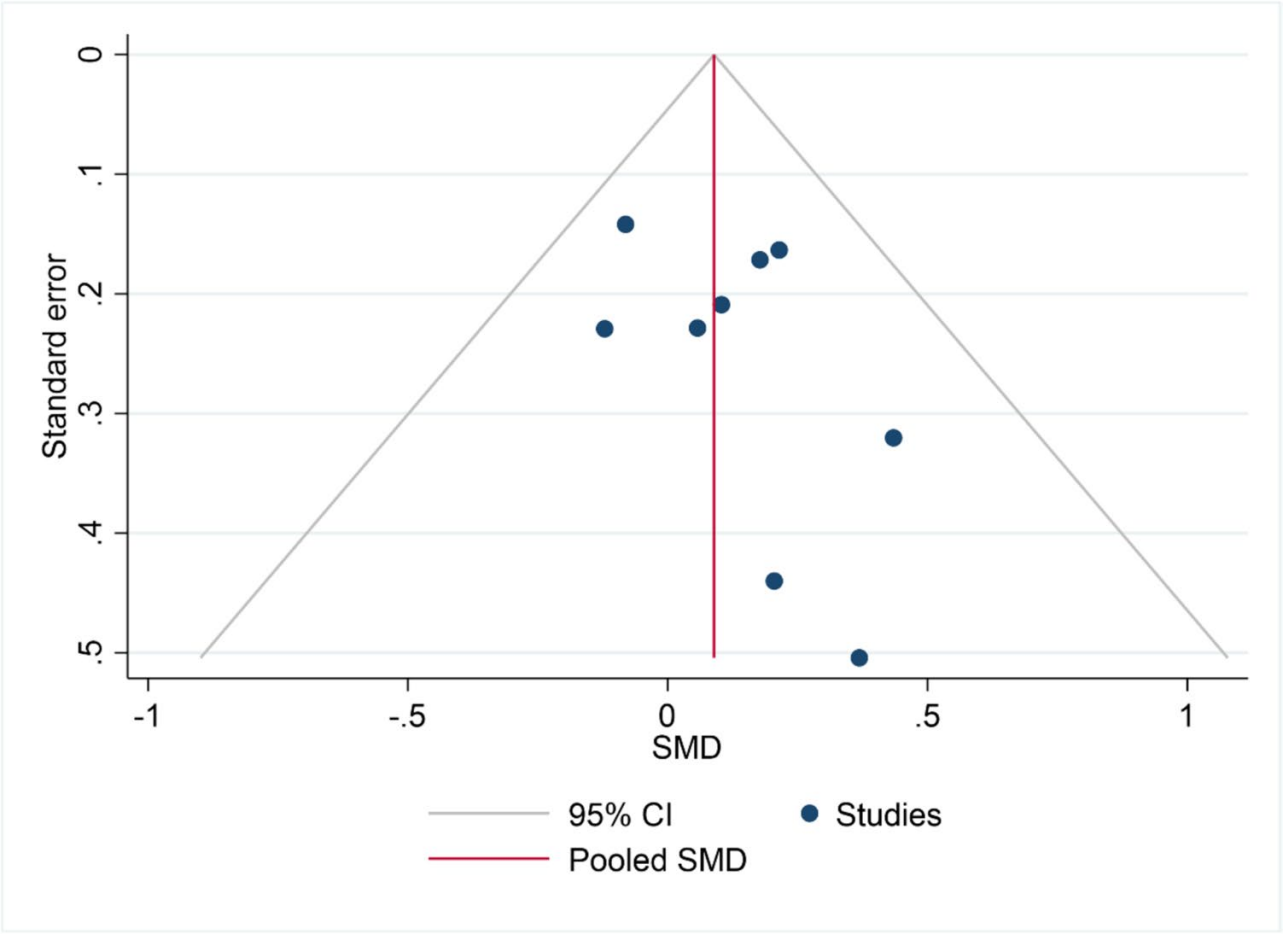
Funnel plot of the studies in the naming analysis at post-treatment. Individual studies are represented by blue dots, with their effect size (x-axis) and standard error (y-axis), together with the combined effect size (red line) with the respective confidence interval’s lower and upper limits (grey lines)

The funnel plot was generated to evaluate small study effects in the meta-analysis of immediate post-treatment naming data, given enough studies were available (Fig. 10) (see the “Reporting Bias Assessment” section). Although the asymmetry at the bottom of the scatter plot might suggest a small-study effect, because the studies with higher standard error (i.e., lower sample size) tend to be more distributed on the right of the combined effect size, this trend is not statistically significant as shown by Egger’s test (bias = 0.91, SE = 0.96; *p* = 0.343).

## Discussion

Primary progressive aphasias are a group of rare, heterogeneous neurodegenerative syndromes characterized by the progressive loss of function of those brain regions involved in language production and comprehension, resulting in the disintegration of patients’ linguistic abilities over time, with devastating implications for patients’ quality of life, work, and social functioning. No pharmacological treatment able to halt or at least slow down the disease course is currently available. To counteract PPA disease progression and ameliorate clinical symptoms, novel, effective therapeutic approaches are urgently needed.

The primary aim of the present meta-analytical study was to evaluate the efficacy of noninvasive brain stimulation methods (NiBS) in improving language functions in PPA patients. The estimated pooled effect was not significant in each of the main analyses conducted, meaning that the change in performance from pre-treatment to post-treatment, short-term and long-term follow-up was not statistically different between the active and sham group in any of the outcomes examined (naming, phonemic, and semantic fluency). One reason for this result could be that the included studies adopted different stimulation modalities (bipolar/multichannel tDCS, rTMS), with different parameters within each modality (Tab. 1), that may not have been equally effective in modulating a complex system like the language network (Hickok & Poeppel, 2007). Most included studies adopted tDCS, but recent meta-analyses on NiBS in Alzheimer’s disease and mild cognitive impairment found a higher effect for rTMS than tDCS both with and without cognitive training (Šimko et al., 2022; Teselink et al., 2021; Yang et al., 2024), a result that could not be tested in the present meta-analysis because of the scarcity of rTMS studies. This evidence could be explained by the different mechanisms of action: compared to tDCS, rTMS penetrates deeper into the skull and stimulates the brain in a more controlled, reliable, and targeted manner; furthermore, rTMS generates action potentials in stimulated neurons whereas tCDS acts at the membrane polarization level without eliciting action potentials. Thus, tDCS may be more effective if associated with language training, as suggested by a higher effect for trained than untrained items reported in a recent Cochrane review (Roheger et al., 2024). Notably, four out of ten included studies did not deliver SLT, which could have further contributed to the overall result.

Regarding stimulation protocols, five-to-ten tDCS or rTMS sessions may not be sufficient to induce substantial neuromodulatory effects and appreciate significant differences between the experimental conditions; indeed, in the studies that adopted more than ten sessions (de Aguiar et al., 2020a, 2020b; Huang et al., 2023; Pytel et al., 2021; Wang et al., 2022), we found larger effect sizes on average. Furthermore, the same protocols of stimulation were applied irrespective of the PPA variant, therefore disregarding the different neuropathological alterations of PPA forms that might benefit from more personalized approaches. The area targeted in most studies was the left inferior frontal one, which is predominantly damaged non-fluent patients; these patients, notably, also represented the majority of the sample (112 out of 229 patients). Each PPA variant could be treated more effectively by identifying the language hubs more spared in terms of local activity and connectivity. Another strategy that has been suggested, on the contrary, is the stimulation of the loci of atrophy in each variant (Tsapkini et al., 2018). The study by Pytel and collaborators (2021) was the only one adopting a personalized approach that determined the most meaningful target for each patient after assessing the language changes with a single stimulation session; according to this method, the best stimulation site for most non-fluent patients was indeed the left inferior frontal gyrus. Albeit the best strategy for their identification is yet to be established, subject- or variant-specific stimulation targets are encouraged in future studies.

Of note, there was substantial heterogeneity in the type of speech-language therapy intervention that was administered during NiBS. For instance, some studies adopted a spelling-based treatment approach (de Aguiar et al., 2020a, 2020b; Wang et al., 2022), whereas another study opted for a modified version of constraint-induced language therapy (CILT) (Nissim et al., 2022). Some SLT protocols could exert greater therapeutic effects than others when coupled with NiBS, depending on the language and/or cognitive process involved. However, while there is evidence for the superior effectiveness of high-intensity treatments of post-stroke aphasia on language recovery, the knowledge about what therapy works best for patients with specific patterns of linguistic impairment and/or lesion location is still insufficient (Fridriksson & Hillis, 2021).

Not least, except for two studies (Hosseini et al., 2019; McConathey et al., 2017), the stimuli did not differ between time points (pre-, post-treatment, and follow-ups), which could have led to bias in the analyses due to practice effects and caused an overall null effect.

However, a statistically significant and moderate effect size emerged in the naming analysis at 2–3 months follow-up for the parallel group, where for example de Aguiar et al., (2020a, 2020b) reported an increase in naming accuracy equal to 14% in the active group versus 1% in the sham group on average. Notably, a similar trend is observed in the semantic fluency analysis, where combined effects increase over time (immediate post-treatment, 2–6 weeks, 2–3 months), although not significant. These results might be framed in the context of a protective effect able to slow down the disease progression, rather than sharply improving language functions at post-treatment.

Interestingly, the pooled effects for parallel studies were systematically higher than those for the crossover studies. This difference could be ascribed to the considerable disparity in sample size: smaller studies may not have sufficient power to detect a modest intervention effect. Moreover, a poorer methodological quality in the crossover studies could have contributed to this difference: an inadequate washout period, for example, may have dampened the difference across experimental conditions because of carryover effects. Two crossover studies (Ferrucci et al., 2018; Roncero et al., 2017) adopted a 2-month washout, probably not entirely sufficient to avoid carryover (Tsapkini et al., 2018). Moreover, of the three studies with a lower risk of bias (Benussi et al., 2020; Huang et al., 2023; Pytel et al., 2021), two were parallel studies and one adopted a mixed design.

To the best of our knowledge, this is the first meta-analysis on placebo-controlled, randomized clinical trials that applied NiBS protocols on PPA. The results are in contrast with those of previous meta-analyses on the same topic, which found moderate or large effect sizes (Byeon et al., 2020; Nissim et al., 2020). If on one hand this discrepancy could be merely explained by the higher number of studies available in this review, the results in the previous meta-analyses were limited by the lack of the sham condition in some of the included studies, which does not allow for accurate establishment of the real additional effect of stimulation. Very recently, Roheger and colleagues (2024) published a Cochrane review that included in part the same studies of our paper, but they did not perform a quantitative synthesis of results because of the evaluation of insufficient data and high heterogeneity. Although we agree on the preliminary nature of the research in this domain, we think that our quantitative approach can be framed in the context of a higher number of studies available (specifically the ones, with notable sample sizes, conducted by Benussi and colleagues (2020) and by Huang and collaborators (2023)). In this sense, our work can be seen as complementary to the thorough review conducted by Roheger and collaborators, adopting a quantitative approach that gives an immediate readout of the state of the art of the literature. It is also important to note that we tried to mitigate the effect of task heterogeneity by carrying out different analyses for different language tasks (naming, semantic fluency, phonemic fluency), on the basis of their reliance on partially distinct neural processes (Biesbroek et al., 2021).

The sensitivity analyses for the naming outcome showed that the combined effect of the studies that coupled NiBS with SLT was slightly higher than the overall effect at each time point, although it did not reach the significance threshold. For phonemic and semantic fluency, the studies were not enough to perform the analysis. This partially goes in the direction of our hypothesis that, when coupled with active training like SLT, NiBS protocols could be more effective on language functions than when used as stand-alone treatments (Neri et al., 2021). However, given the limited number of studies, further evidence is necessary to confirm this hypothesis.

The present study has some limitations to consider. Given the subthreshold number of studies, the meta-analyses of follow-up data on phonemic fluency could not be carried out. The same limitation also prevented us from performing the assessment of the risk of bias across studies via funnel plots in each analysis, except for the naming analysis on immediate post data. Furthermore, because of the scarcity of the studies available, meta-regression or subgroup analyses could not be applied in any of the analyses performed, and only a description of the results was reported for the risk of bias subgroups. Indeed, in the object naming analyses, only one study performed rTMS at post-treatment (Pytel et al., 2021) and follow-up(s) (Huang et al., 2023), while for the other outcomes, only tDCS studies were available. Similarly, there was not a sufficient number of studies reporting MMSE scores. Regarding the PPA variant, the considerable heterogeneity between studies made subgroup comparisons difficult to perform. The investigation of heterogeneity via meta-regression would have given a hint on the role of variables (disease severity, PPA variant, stimulation method) that presumably had an impact on results. Future studies with larger sample sizes are critically needed to disentangle the real impact of brain stimulation on PPA. Indeed, the fact that many studies were underpowered could have contributed to the overall null results: given the high variability among PPA patients, much larger sample sizes are probably required to spot significant differences. Many studies were also affected by high susceptibility to bias, so the final results should be interpreted with caution. It is also important to note that the findings of this study refer to a specific subset of language tasks (naming and fluency). Other language tasks (e.g., connected speech) or non-language effects (other cognitive domains, psychological factors, quality of life) could not be examined. It might therefore be interesting for future studies to include these aspects that may be relevant in patients with PPA, as shown in some of the included studies (Cotelli et al., 2014; Pytel et al., 2021).

### Conclusions

To our knowledge, this was the first meta-analysis of randomized controlled trials applying NiBS on PPA patients. The overall null results, in light of the limitations discussed above, have to be considered preliminary and cry out for future research in the field, especially large RCTs adopting rTMS as a neuromodulatory technique and testing variant-specific stimulation protocols that may be more effective on a heterogeneous group of disorders like PPAs. Other endeavors towards personalization of therapies (e.g., according to disease severity) are strongly encouraged. Also considering longer treatment periods (e.g., more than 15 sessions) could be an important step forward in the future. At a deeper unit of analysis, interesting results come from stratifying by study design, where parallel trials outperformed crossover trials, and from a longitudinal perspective, that shows an increase over time in estimated effect sizes. Moreover, the combined effect for the few studies that coupled NiBS with SLT was slightly higher than the overall effect at each time point, although not significant, suggesting that the association between NiBS and SLT is worth further exploring in future literature.

## Supplementary Information

Below is the link to the electronic supplementary material.

Supplementary file1 (DOCX 41 KB)

## Data Availability

The data that support the findings of this study are available from the corresponding author upon request.
